# Modulation of αv integrins by lebecetin, a viper venom-derived molecule, in experimental neuroinflammation and demyelination models

**DOI:** 10.1038/s41598-024-73259-1

**Published:** 2024-09-27

**Authors:** Nour-elhouda Neili, Zaineb AbdelKafi-Koubaa, Jed Jebali, Khouloud Kaidi, Ghada Sahraoui, Melika Ben Ahmed, Najet Srairi-Abid, Naziha Marrakchi, Raoudha Doghri, Ines ELBini

**Affiliations:** 1 Laboratory of Biomolecules, Venoms and Theranostic Applications (LR20IPT01), Pasteur Institute of Tunis, University of Tunis, El Manar, Tunis, Tunisia; 2 Research Laboratory of Precision Medicine/Personalized Medicine and Oncology Investigation (LR21SP01), Saleh Azaiez Institute, Tunis, Tunisia; 3 Faculty of Medicine of Tunis, University of Tunis, El Manar, Tunis, Tunisia; 4 Laboratory of Transmission, Control and Immunobiology of Infections (LR16IPT02), Pasteur Institute of Tunis, University of Tunis, El Manar, Tunis, Tunisia

**Keywords:** Neuroinflammation, Demyelination, Integrins, Astrocytes, Oligodendrocytes, Cuprizone, Neuroscience, Physiology

## Abstract

Several neurodegenerative diseases, such as multiple sclerosis and Parkinson’s disease, are linked to alterations in myelin content or structure. Transmembrane receptors such as integrins could be involved in these alterations. In the present study, we investigated the role of αv-integrins in experimental models of neuroinflammation and demyelination with the use of lebecetin (LCT), a C-lectin protein purified from *Macrovipera lebetina* viper venom, as an αv-integrin modulator. In a model of neuroinflammation, LCT inhibited the upregulation of αv, β3, β5, α5, and β1 integrins, as well as the associated release of pro-inflammatory factor IL-6 and chemokine CXCL-10, and decreased the expression of phosphorylated NfκB. The subsequent “indirect culture” between reactive astrocytes and oligodendrocytes showed a down-regulation of αv and β3 integrins versus upregulation of β1 one, accompanied by a reduced expression of myelin basic protein (MBP). Treatment of oligodendrocytes with LCT rectified the changes in integrin and MBP expression. Through Western blot quantification, LCT was shown to upregulate the expression levels of PI3K and p-mTOR while downregulating expression levels of p-AKT in oligodendrocytes, suggesting the neuroprotective and pro-myelinating effects of LCT may be related to the PI3K/mTor/AKT pathway. Concomitantly, we found that LCT promoted remyelination by tracking the increased expression of MBP in the brains of cuprizone-intoxicated mice. These results point to an involvement of integrins in not only neuroinflammation but demyelination as well. Thus, targeting αv integrins could offer potential therapeutic avenues for the treatment of demyelinating diseases.

**Supplementary Information:**

The online version contains supplementary material available at 10.1038/s41598-024-73259-1.

## Introduction

Dysfunctions affecting central nervous system (CNS) myelin contribute to a substantial and expanding array of neurological disorders. This spectrum encompasses prevalent myelin diseases as well as rare genetic conditions^1^. Demyelinating diseases are a type of neurological disorder characterized by progressive damage to the myelin sheath in the nervous system. Indeed, several neurodegenerative diseases have been associated with changes to myelin content or structure in white matter^2^. This includes diseases commonly associated with demyelination such as multiple sclerosis (MS)^3^, along with other diseases that have more gray matter changes such as Huntington’s disease, motor neuron disease, amyotrophic lateral sclerosis, and Parkinson’s disease^4^. The association between these disease pathologies and their impacts on myelin can occur through different mechanisms. Inflammation, oxidative stress, alterations in cellular metabolism, and changes in the extracellular matrix may collectively contribute to the process of demyelination^5^. These factors are hallmarks of certain demyelinating diseases including MS. In this context, neuroinflammation is primarily driven by activated microglia and astrocytes, releasing a variety of inflammatory mediators^6^. Additionally, the infiltration of immune cells sensitized to myelin proteins acts as a central catalyst, not only promoting demyelination but also intensifying localized neuroinflammation, leading to subsequent gliosis and axonal degeneration; this ultimately culminates in the disruption of neuronal signaling^7,8^.

Traditionally, demyelination research has predominantly centered on oligodendrocytes, the primary cells affected by this phenomenon. However, recent literature highlights an evolving understanding of the involvement of other glial cells, particularly astrocytes. These cells not only play a role in myelination but also contribute to the regulation of neuroinflammation during the progression of demyelinating diseases^9,10^.

Among other emerging cellular effectors involved in myelination, integrins seem to be promising candidates for exploration. Their close involvement in the process of myelination positions them as noteworthy contributors to the intricate mechanisms at play in these pathologies. Integrin proteins are ubiquitous, heterodimeric, transmembrane glycoprotein receptors that primarily act as signaling proteins in mammals^11,12^. Each consists of an α- and β-subunits which are bound in a noncovalent complex with the ligand-binding site at the interface. Integrins act as adhesion receptors, with the unusual ability to signal in both directions across the plasma membrane^13^. These receptors have previously been reported to primarily cause or contribute to a number of diseases and are regarded as prominent targets for treatment^12^.

The present study aims to determine the potential role of integrins in neuroinflammation and demyelination, the latter was obtained through the indirect interactions between reactive astrocytes and oligodendrocytes. In oligodendrocytes, engagement of the integrin receptor family initiates intracellular signaling cascades that are crucial to cell proliferation, survival, and maturation^14^. Moreover, previous data have shown that the expression of integrins by oligodendrocytes is not only developmentally regulated, but is affected by the nature of the surrounding environment^15^. Indeed, integrins are expressed in developing stages of demyelinating diseases but also in adult tissues, being involved in pathological conditions^16,17^. In MS pathology, an increased interest in α4β7 and α4β1 has presumably come from the clear benefit to patients from prescribed drugs, such as natalizumab, which specifically targets the α4 integrin subunit^17,18^. However, the engagement of other types of integrins in demyelination has not been examined. To identify these integrins and elucidate their mechanisms of action in demyelination, we resorted to the use of a natural biomolecule (lebecetin; LCT) purified from the venom of Tunisian viper *Macrovipera lebetina transmediterranea* (*M. lebetina*), as an identification tool. This biomolecule is known to target αv-containing integrins^19,20^. Proven to exhibit an anti-inflammatory effect, LCT modulated LPS-induced inflammatory cytokine production in human THP-1-derived macrophages^21^. The following work delineates the impact of modulating target integrins in neuroinflammation and demyelination, shedding light on unexplored facets of their contribution to these processes.

## Results

### Integrin involvement in cellular models of neuroinflammation and demyelination

#### Effect of lebecetin on glial cell viability

**Fig. 1:**
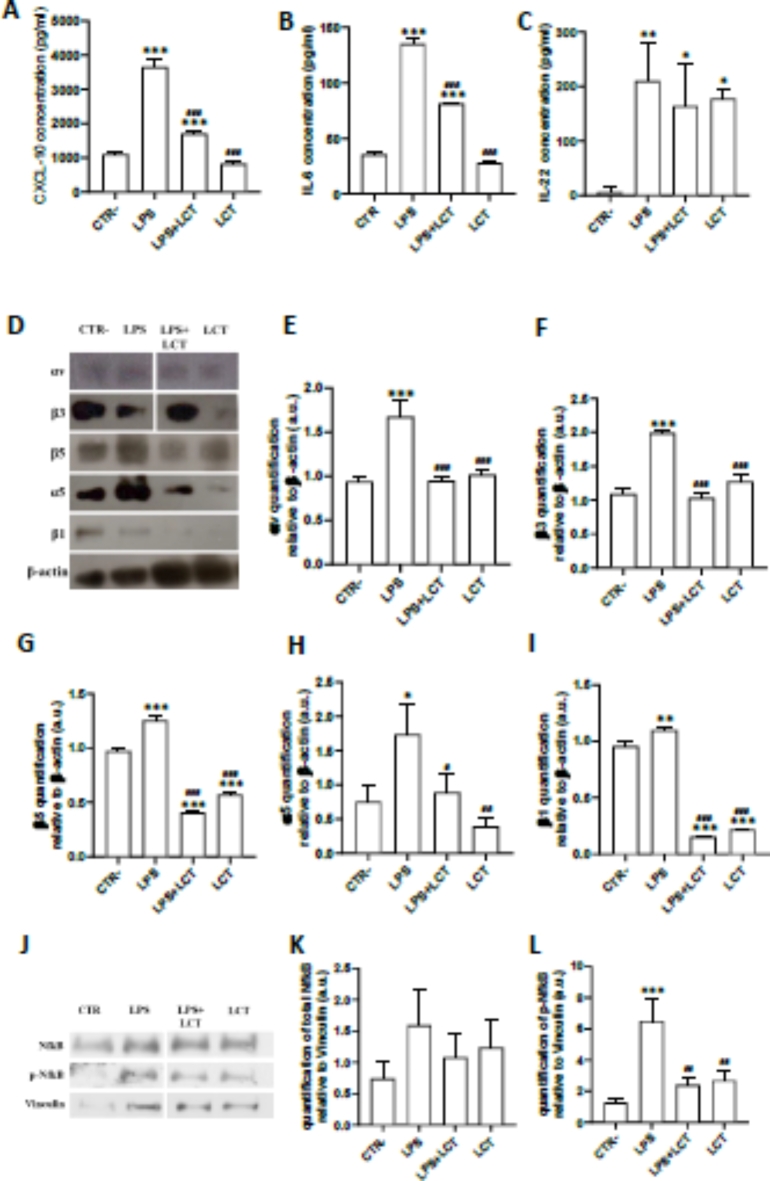
Lebecetin inhibited LPS-induced secretion of chemokine (**A**) CXCL10, and cytokines (**B**) IL-6 and (**C**) IL-22 via Classic ELISA in astrocytes. (**D**) Lebecetin modulates integrin expression in reactive astrocytes determined by Western Immunoblotting; the quantification of (**E**) αv, (**F**) β3, (**G**) β5, (**H**) α5, and (**I**) β1 integrin subunits was done after normalization with β-actin. (**J**) Lebecetin decreased the LPS-induced activation of NfκB p65, by Western Immunoblotting. The quantification of (**K**) total and (**L**) phosphorylated NfκB in reactive astrocytes was done after normalization with vinculin. Gels were cropped to remove repeated conditions for clarity. CTR: negative controls; LPS: cells treated with LPS; LPS + LCT: cells pre-treated with LCT then stimulated with LPS; LCT: cells treated with LCT. *p < 0.05, **p < 0.01, ***p < 0.001 compared to CTR−. ^#^*p* < 0.05, ^##^*p* < 0.01, ^###^*p* < 0.001 compared to LPS.

Lebecetin is a natural molecule derived from viper venom; therefore, assessing its cytotoxicity is the initial step in determining the optimal dose with no apparent toxicity, we treated astrocytes with a range of concentrations (50–3000 nM) for 24 h. Our results showed that, up to a dose of 200 nM, LCT did not affect the viability of the glial cell line used. A slight cytotoxicity was first observed at 400 nM (Fig. 1A, Supplementary data), this cytotoxicity persisted in a dose-dependent manner with an IC50 of 1000 nM, and the highest toxicity levels were observed at a concentration of 3000 nM.

To further ascertain that LCT does not affect cell viability up to a concentration of 200 nM, both astrocytes and oligodendrocytes were treated with 100 and 200 nM of LCT respectively (Fig. 1B,C, Supplementary data). After 24 h, both concentrations did not affect the cell viability in both cell lines. The following experiments were carried out with the 100 nM concentration of LCT.

#### Integrin modulator inhibited the release of IL-6 and CXCL-10 and regulated signaling pathway in reactive astrocytes

Since LCT exhibits anti-inflammatory activity in macrophage cells^21^, we decided to investigate its effects on astrocytes in relation to integrin. Astrocytes were stimulated with LPS to induce an inflammatory reaction, 24 h following treatment, significant levels of chemokine CXCL-10 (Fig. 1A), cytokine IL-6 (Fig. 1B), and cytokine IL-22 (Fig. 1C) were detected through ELISA quantification. In accordance with our hypothesis, results showed that pre-treatment with LCT modulates the release of pro-inflammatory cytokines IL-6 as well as the chemokine CXCL10, consequently modulating the astrocytic inflammatory response, yet increased the release of IL-22 in a similar manner to that of LPS.

Concomitantly, visualization of western immunoblotting results on treated astrocytes showed that in the presence of LPS, the expression of integrin subunits αv (Fig. 1D,E), β3 (Fig. 1D,F), β5 (Fig. 1D,G), α5 (Fig. 1D,H), and β1 (Fig. 1D,I) was upregulated compared to negative controls. In contrast, the expression of these subunits decreased in cells pre-treated with LCT (Fig. 1D–I).

To further characterize whether the modulation of integrins inhibited neuroinflammation in reactive astrocytes, the Nuclear factor-κB (NfκB), a pivotal transcription factor particularly involved in the immune response, was investigated in C8-D1A cells. Western blot analysis showed that after 24 h, in the conditions LPS, LPS + LCT, and LCT, the expression of total NFκB is slightly upregulated compared to CTR−, but statistically non-significant (Fig. 1J,K). However, phosphorylated NfκB was significantly upregulated in reactive astrocytes (Fig. 1J,L), while treatment of cells with LCT induced a significant decrease in the levels of phosphorylated NfκB, that reached a basal level when the protein was combined with LPS (Fig. 1J,L). Indeed, For the LPS + LCT and LCT conditions, the results are similar to a negative control without affecting the basal expression (Fig. 1J,L).

Interestingly, our results showed the capability of LCT to modulate the expression of integrins associated with the inhibition of the release of pro-inflammatory cytokines in reactive astrocytes which is mediated by the NfκB pathway. Therefore, LCT inhibited LPS-induced inflammatory response by inhibiting the NfκB pathway along with αvβ3 and αvβ5 integrins in C8-D1A cells. Nevertheless, we cannot exclude that other cellular effectors may be involved in the biological activity of LCT in reactive astrocytes.

#### Alterations in integrin expression levels affected MBP expression in the astro-oligodendroglial indirect “co-culture” model

**Fig. 2:**
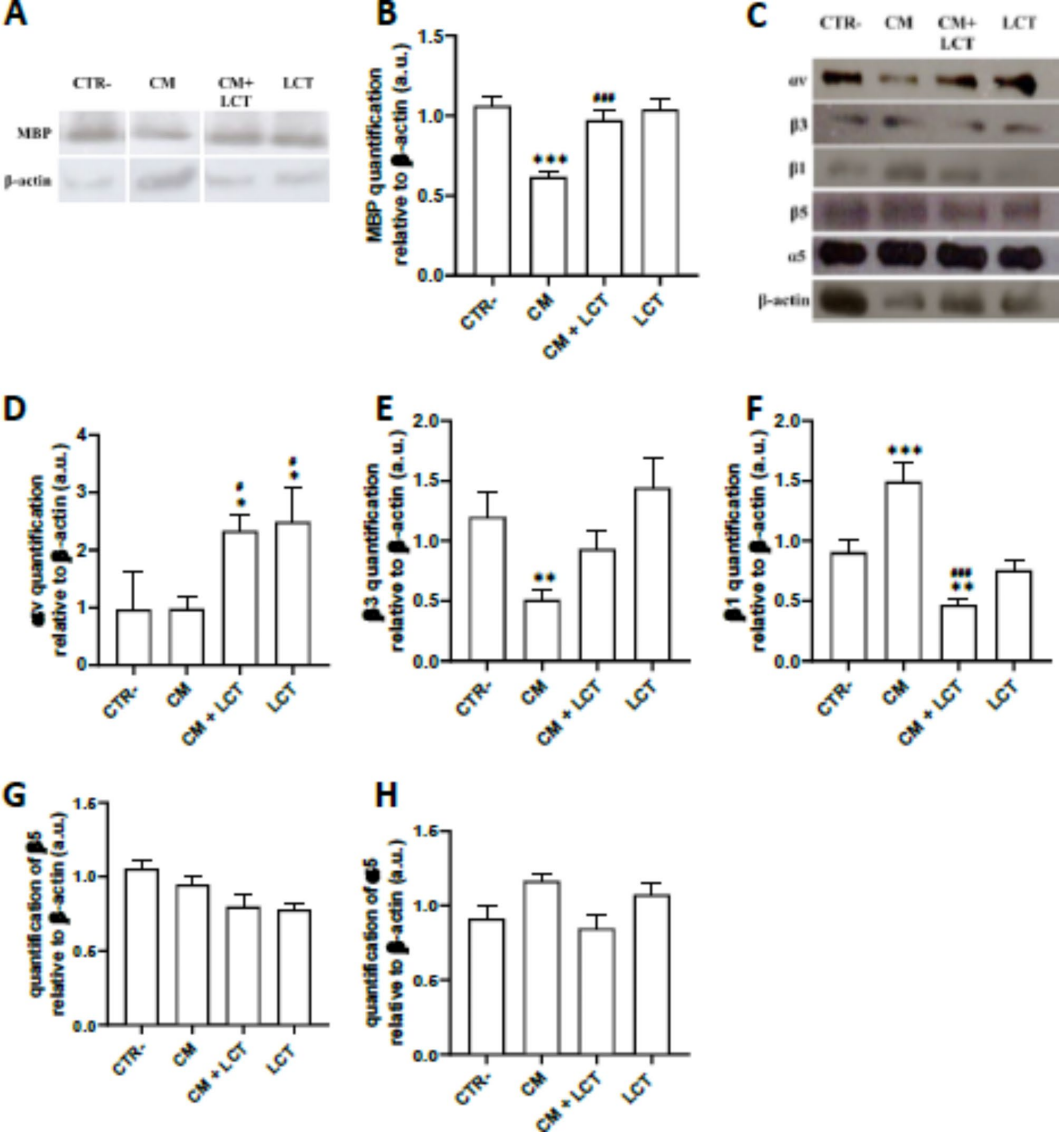
(**A**,** B**) Lebecetin increased the expression level of myelin Basic Protein (MBP); the quantification of MBP in oligodendrocytes treated with LCT and/or conditioned media (CM) was done by Western Immunoblotting. (**C**) Lebecetin modulates integrin expression in stimulated oligodendrocytes determined by Western Immunoblotting; Quantification of (**D**) αv, (**E**) β3, (**F**) β1, (**G**) β5, and (**H**) α5 integrin subunits in the oligodendrocytes was done after normalization with β-actin. Gels were cropped to remove repeated conditions for clarity. CTR: negative controls; CM: cells treated with conditioned media of reactive astrocytes; CM + LCT: cells pre-treated with LCT then with conditioned media of reactive astrocytes; LCT: cells treated with LCT. *p < 0.05, **p < 0.01, ***p < 0.001 compared to CTR−. ^#^*p* < 0.05, ^##^*p* < 0.01, ^###^*p* < 0.001 compared to CM.

To better understand the alterations brought on by reactive astrocytes on myelinating oligodendrocytes, an indirect and unilateral “co-culture” model was established between both cell lines. This system was established by stimulating oligodendrocytes with astrocyte’s LPS conditioned media, oligodendrocytes were either treated with conditioned media (CM) or pre-treated with LCT followed by conditioned media (CM + LCT). To assess myelination, Myelin Basic Protein (MBP) expression was examined in cultured oligodendroglial cells through quantitative analysis by western immunoblotting. Result analysis showed that LCT did not alter MBP expression, maintaining a similar level of expression as that of negative controls (Fig. 2A,B). An increase in αv (Fig. 2C,D) and β3 (Fig. 2C,E) integrin subunits was also observed, along with a down-regulation of β1 expression levels (Fig. 2C,F) compared to negative control. However, no significant variation was observed for the protein expression of α5 (Fig. 2C,G) and β5 (Fig. 2C,H). In contrast, compared to negative controls, MBP expression is significantly decreased in CM-treated astrocytes (Fig. 2A,B), this is accompanied by a decrease in the expression of integrin subunits αv and β3 (Fig. 2C,E), and an increase in β1 (Fig. 2C,F). Treatment with LCT (CM + LCT) significantly restored MBP levels compared to CM, bringing MBP levels to a similar range as those in the CTR− group. Additionally, LCT treatment markedly increased the expression levels of both αv integrins (Fig. 2C,D) and β3 integrins (Fig. 2C,E). In contrast, integrin β1 (Fig. 2C,F) levels were significantly decreased in LCT-treated oligodendrocytes compared to those treated with CM alone.

**Fig. 3:**
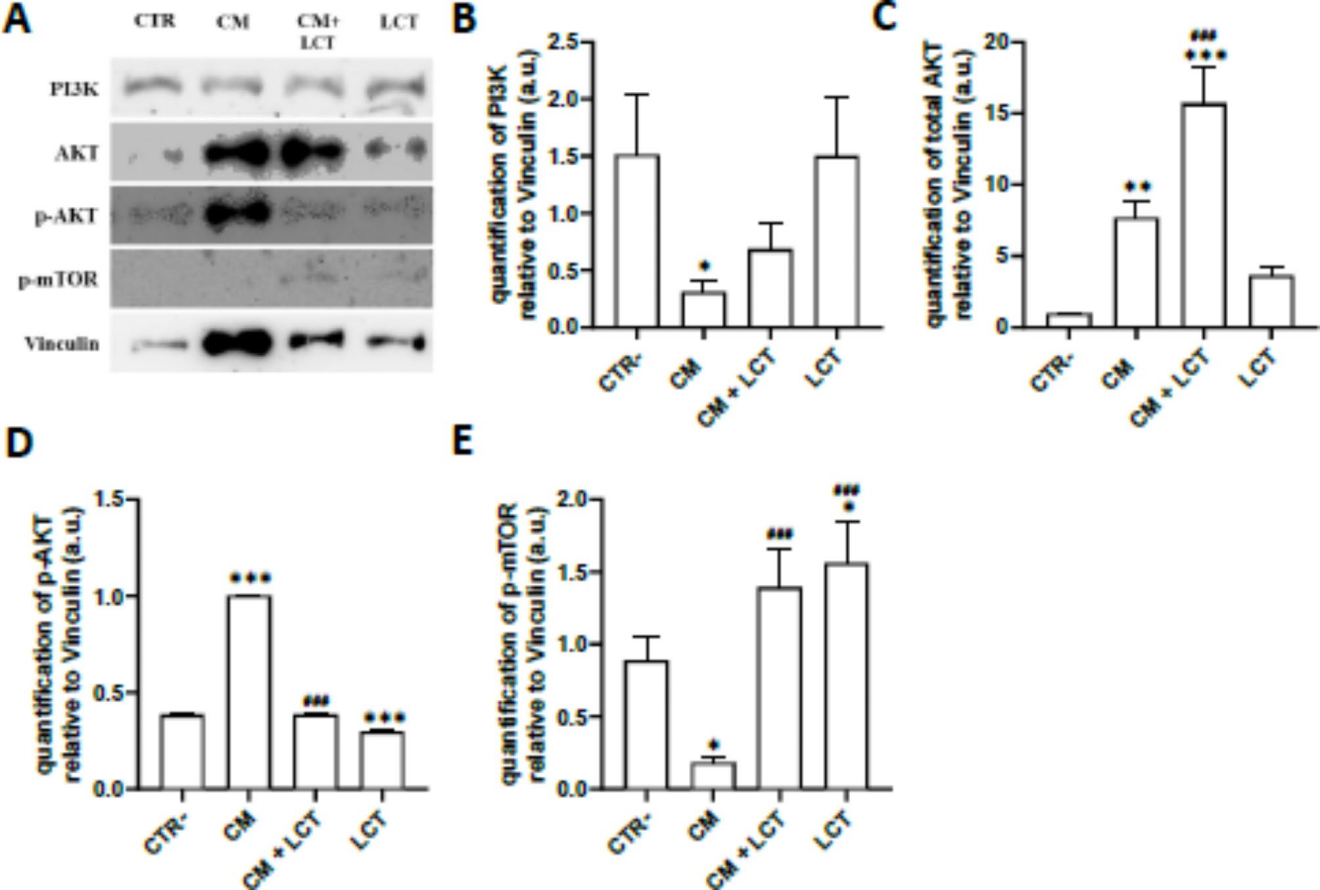
(**A**) Lebecetin regulates the PI3K/AKT/mTor pathway in oligodendrocytes. The quantification of (**B**) PI3K p85, (**C**) total and (**D**) phosphorylated AKT, and (**E**) phosphorylated mTOR in oligodendrocytes treated with LCT and/or CM was done after normalization with Vinculin by Western Immunoblotting. Gels were cropped to remove repeated conditions for clarity. CTR: negative controls; CM: cells treated with conditioned media of reactive astrocytes; CM + LCT: cells pre-treated with LCT then with conditioned media of reactive astrocytes; LCT: cells treated with LCT. *p < 0.05, **p < 0.01, ***p < 0.001 compared to CTR−. ^#^*p* < 0.05, ^##^*p* < 0.01, ^###^*p* < 0.001 compared to CM.

To further characterize whether the modulation of integrin enhanced myelination, particularly the production of myelin proteins, which is associated with the regulation of signaling pathway PI3K/AKT/mTOR in oligodendrocytes (Fig. 3A). The results showed that CM stimulation induced the upregulation of both protein kinase B (AKT) and its phosphorylated form (p-AKT) (Fig. 3A,C,D) and a downregulation of Phosphoinositide 3-kinases (PI3K) (Fig. 3A,B) and the phosphorylated form of mechanistic target of Rapamycin (p-mTOR) (Fig. 3A,E). Pre-treating with LCT reversed the effect of CM on p-AKT and p-mTOR, significantly downregulating pAKT and upregulating p-mTOR expression compared to CM-treated oligodendrocytes. However, pretreatment with LCT only slightly increased PI3K levels (non-significant), and significantly increased total AKT expression levels compared to CM-treated oligodendrocytes.

We showed that the conditioned media from astrocytes reduce the expression of MBP in oligodendrocyte cells by altering integrin expression, which affects the PI3K/AKT/mTOR signaling pathway. Pre-treatment with LCT modified the expression of the αvβ3 integrin and maintained MBP levels through the AKT/mTOR pathway, thereby influencing oligodendrocyte function.

### Confirmation of integrin involvement in an animal model of demyelination

Following in vitro experiments, the present study explored whether integrin involvement could influence demyelination in C57BL/6 female mice treated with cuprizone, a model reflecting this condition. To investigate, cuprizone was administered to mice for 8 weeks, closely followed by treatment with LCT, an integrin modulator.

#### Integrin-modulator improved cognitive and motor performance in cuprizone mouse model

**Fig. 4:**
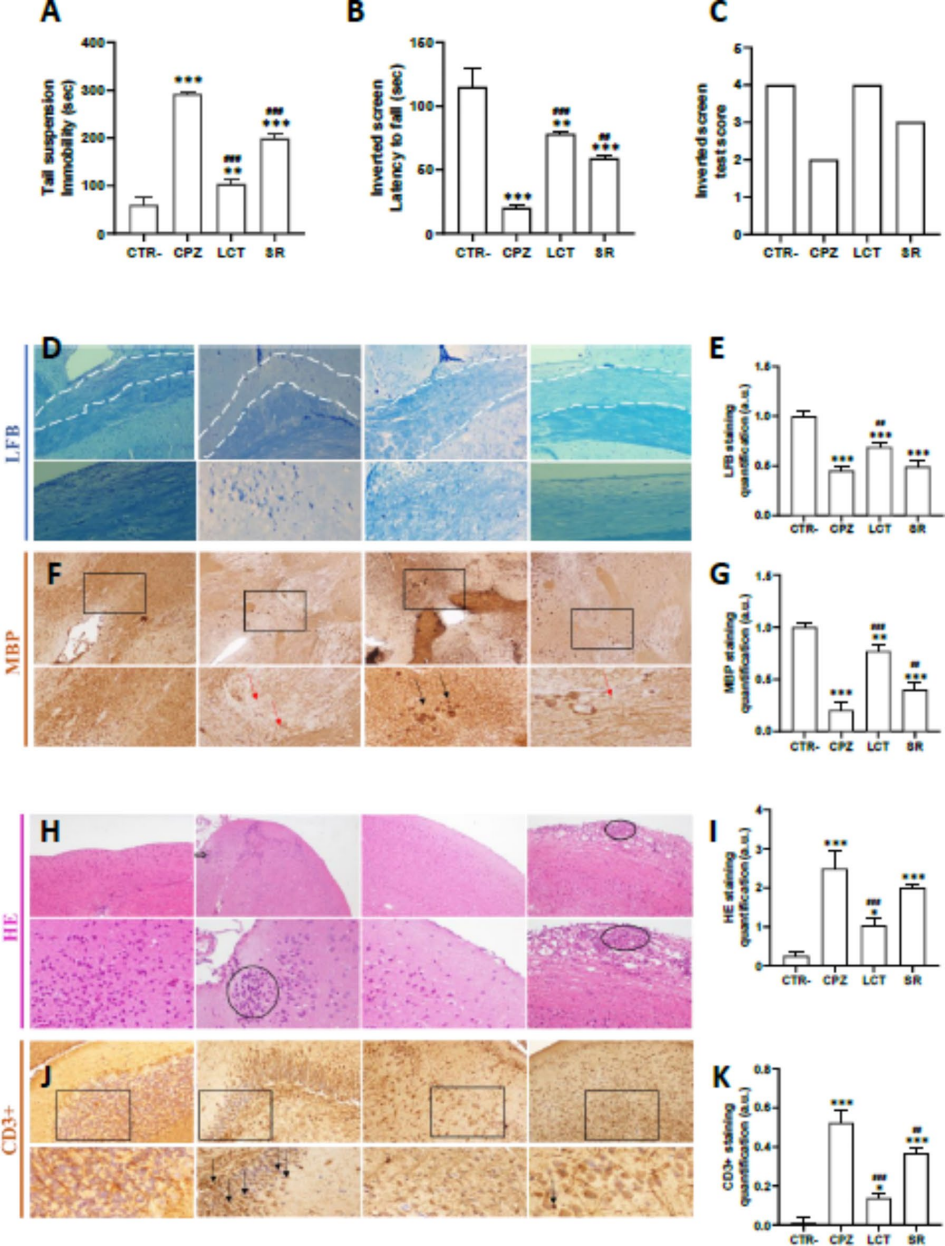
(**A**) Illustration of the total time spent immobile (in seconds) in the the tail suspension test for all treatment groups. (**B**) Illustration of the latency to fall from the screen (in seconds) and (**C**) subsequent test scores in the inverted screen test for all treatment groups. (**D**,**E**) Quantification of myelin in the corpus callosum of a C57BL/6 mice given cuprizon for 6 weeks and treated with LCT, showing an area with de- and re-myelination using Luxol fast blue staining technique. The corpus callosum is delineated with 2 parallel dotted lines. (**F**,**G**) Immunohistological staining and quantification of monoclonal mouse anti-myelin basic protein (MBP) from the corpus callosum of a C57BL/6 mice given cuprizon for 6 weeks and treated with LCT. Black arrow: staining for MBP, as a measure of myelinization while the red arrow indicates a demyelinated area. (**H**,**I**) Hematoxylin and eosin staining and quantification, and (**J**,**K**) Immunohistological staining and quantification of monoclonal mouse anti-CD3 + from the brain sections of a C57BL/6 mice given cuprizon for 6 weeks and treated with LCT. (Microscopic magnification: ×10; ×40). *CTR* control group, *CPZ* mice treated with cuprizon, *LCT* mice treated with LCT after the cuprizon-administration period; SR: mice who have undergone spontaneous remyelination after the cuprizone-administration period. *p < 0.05, **p < 0.01, ***p < 0.001 compared to CTR−. ^#^*p* < 0.05, ^##^*p* < 0.01, ^###^*p* < 0.001 compared compared to cuprizone-treated group.

To verify the changes in behavior caused by cuprizone, all control and treated animals underwent identical testing procedures, conducted in the same environment and conditions by the same researcher. The results of the tail suspension test showed a significant increase in the duration of immobility for cuprizone-treated mice compared to the control group (Fig. 4A), which may indicate depression-related behavior induced by the administration of cuprizone. Meanwhile, the experimental group treated with LCT following the cuprizone administration period showed a decrease in the duration of immobility, which is even more pronounced compared to the spontaneous remyelination (SR) group. The inverted screen test results followed a similar profile, wherein mice treated with cuprizone had a significantly shorter fixation period than the control group, showing that cuprizone-induced muscle fatigue in mice (Fig. 4B). Meanwhile, mice treated with LCT following the cuprizone administration period exhibited a longer fixation period. The difference was statistically relevant compared to the control and the spontaneous remyelination groups. For the inverted screen test scores (S), the lowest score was attributed to the cuprizone group (S = 0.5). In contrast, the highest score was attributed to the LCT-treated group (S = 3), and the spontaneous remyelination group received an acceptable score (S = 2.2) (Fig. 4C). These results demonstrated significant muscle weakness in cuprizone-treated mice, further consolidating the notion of motor dysfunctions due to cuprizone-induced demyelination. This dysfunction seems to be attenuated by the administration of LCT.

#### Integrin-modulators suppressed cuprizone-induced neuro-inflammation and improved remyelination in brain sections

Sections of the brain from all experimental groups were stained to visualize both demyelination and inflammation. Histochemical staining with Luxol Fast Blue (LFB) was used to visualize myelin in nerve tissue. Figure 4 roughly illustrates the level of demyelination in the *Corpus Callosum* (CC) region of the brain of treated mice. A severe myelin loss is apparent in the brain of cuprizone-treated mice (CPZ) compared to the control group (CTR) (Fig. 4D,E). In contrast, a significantly higher amount of myelin is observed in the brains of mice treated with LCT after the 6-week cCuprizone administration period when compared to mice that were left untreated to undergo spontaneous remyelination (SR) (Fig. 4D,E). To determine the extent of demyelination, myelin was evaluated by MBP immuno-labeling. The quantification of myelin was further determined by immunohistological staining of myelin proteins in the CC using monoclonal mouse anti-myelin basic protein (MBP) primary antibodies. A decrease in MBP expression was found in the brains of CPZ mice compared to controls (Fig. 4F,G) MBP levels were significantly improved by LCT treatment when compared to the SR group (Fig. 4F,G).

A second staining on brain sections from all treatment groups was performed using HE, to assess neuroinflammation. Staining in the brains of cuprizone-treated mice (CPZ) showed considerable inflammatory cell infiltration (Fig. 4H,I), which was in keeping with expectations. Conversely, treatment with LCT significantly decreased inflammatory cell infiltration in the LCT group compared to both the CPZ and SR groups (Fig. 4H,I). To identify the types of immune cells that infiltrate brain tissue of the demyelination model, we carried out an immunohistochemistry analysis to highlight the expression levels of TCD3 as a marker of infiltration of T cells (Fig. 4J). The quantitative results are also presented in Fig. 4K. The expression of LTCD3 cells in the brain was significantly increased in the CPZ and SR groups, while they decreased in the group treated with LCT when compared with the positive groups (Fig. 4J,K).

Our in vivo studies indicate that extended exposure to cuprizone induces molecular alterations in the brain, resulting in demyelination, as evidenced by considerable myelin loss and decreased MBP expression in cuprizone-treated mice. Furthermore, administering cuprizone at a dose of 300 mg/kg body weight caused brain inflammation in the mice, linked to an increased expression of LTCD3 cells. Notably, three intracerebroventricular injections of LCT at 1 mg/ml significantly reduced neuroinflammation and decreased CD3 cell expression.

## Discussion

The present study investigates the role of αv-integrins in both neuroinflammation and demyelination. These integrins are targeted specifically by lebecetin, a natural protein purified from viper venom, and used as a proof of concept. This potential involvement of integrins was studied through the use of established cell models, and subsequently validated using an animal model. In this study, two main cellular models were used; the first model aimed to induce inflammation in the cell line of murine astrocytes. Indeed, astrocytes are the most abundant glial cells in the CNS^22^, and are known to establish multiple interactions with other resident cells. These interactions occur in a healthy brain and involve the astrocytic crosstalk with neurons, microglia, oligodendrocytes, and other astrocytes, thus allowing astrocytes to fulfill functions of metabolic and homeostatic maintenance^23^. In neuroinflammatory conditions, these interactions change and may affect the pathological processes that depend on the inflammatory stimuli released by the astrocytes into the milieu^24^. Reactive astrogliosis is associated with increased production of cytokines and other agents that can damage neurons^25^ and oligodendrocyte functions^26^. Thus, with the aim of investigating the role of αv-integrins in demyelination while taking into consideration the crosstalk in the neural environment, we used a second cell model. This model aimed to partially emulate the interaction between astrocytes and oligodendrocytes by culturing oligodendrocytes in the presence of astrocyte-conditioned media. This approach allowed us to study the influence of astrocyte-derived factors on the expression and function of integrins in oligodendrocytes, which are the primary myelinating cells in the central nervous system.

Under the present experimental conditions, LPS-induced activation of astrocytes is characterized by a significant increase in pro-inflammatory cytokine IL-6 and chemokine CXCL10. IL-6 is one of the driving factors behind neuroinflammation-induced neurodegeneration. It is also crucial for the activation of encephalitogenic Th17 cells^27^. Prolonged inflammatory reactions coupled with increased IL-6 levels work in opposition and instead promote the progression of neurodegenerative diseases^28^. In addition to abnormal levels of IL-6 indirectly impacting demyelination by exacerbating the inflammatory response, it is equally important to consider the effects of chemokine CXCL10 as it directly impacts both neuroinflammation and myelination. CXCL10 binds to the cell surface receptor CXCR3, which is expressed on neurons as well as immune and glial cells. CXCL10 was shown to be majorly expressed by reactive astrocytes surrounding active MS lesions^29,30^. Indeed, CXCL10 mRNA is significantly increased during peak disease and reduced during recovery phases in the experimental autoimmune encephalomyelitis (EAE) model^31–33^. Interestingly, Clarner et al. reported the significant involvement of CXCL10 in neuroinflammatory processes associated with oligodendrocyte pathology^34^. Concomitantly, CXCL10 was shown to directly inhibit myelination in vitro, astrocytes with upregulated levels of CXCL10 seemed to impede myelination by inhibiting oligodendrocyte process extension^35^. Furthermore, the addition of CXCL10 protein to normal myelinating cultures led to the reduction of myelinated axons within the culture. These data suggest that astrocyte-derived CXCL10 acts directly on oligodendrocyte maturation and axonal wrapping. It was highlighted in a subsequent study, that CXCL10 dramatically reduces myelin sheath numbers without affecting oligodendrocyte progenitor cells (OPC) proliferation and differentiation, suggesting that it influences oligodendrocyte/axonal interactions, preventing myelin ensheathment^35,36^. The present study showed that the introduction of an integrin-modulator, LCT, to astroglial cells prior to LPS treatment, significantly impacted the levels of pro-inflammatory cytokines released by astrocytes. In addition, reducing the production of pro-inflammatory cytokines and chemokines such as IL-6 and CXCL10 is crucial for regulating and controlling the inflammatory response. Our results suggest that LCT may contribute to controlling excessive inflammation by lowering the levels of these pro-inflammatory cytokines and chemokines. Unfortunately, no existing work has examined the impact of animal venom biomolecules on neuroinflammation at the level of glial cells and, more specifically, astrocytes. Therefore, we compare our results with those from other models; including previous research showing that LCT reduced the levels of the pro-inflammatory cytokines TNF-α, IL-6, and IL-8 while it partially increased LPS-induced secretion of the immunomodulatory cytokine IL-10 in (LPS)-induced THP1 human macrophages^21^. A previous study showed that the disintegrin Trimucrin from the venom of *Trimeresurus mucrosquamatus* decreased the release of pro-inflammatory cytokines TNFα and IL-6 in LPS-activated macrophages^37^.

In addition to the previously mentioned cytokines, IL-22 levels were increased in the presence of LPS as well as LCT, both individually and in combination. IL-22 is a Th-17-linked cytokine that has been correlated to several autoimmune diseases such as inflammatory bowel disease and psoriasis^38^, and yet is barely studied in the context of MS^39^. Depending on the targeted tissue and surrounding environment, IL-22 can contribute to inflammation, chemotaxis, and host defense but also to cell survival, tissue protection, wound healing, and epithelial cell proliferation^40–43^. IL-22, along with IL-17, appear to compromise BBB integrity, potentially influencing MS severity^38,41^. Notably, this secreted IL-22 inhibitory receptor exacerbated the EAE disease course^44^, prompting consideration of whether IL-22 itself possesses anti-inflammatory functions in EAE.

On the other hand, the reactive state of astrocytes was accompanied by an upregulation of integrin subunits, as observed through western immunoblotting. The introduction of LPS induced the upregulation of integrin subunits αv, β3, β5, α5, and β1. These results correlate with a number of studies exploring neuroinflammation. During active astrogliosis, astrocyte migration requires cell cytoskeletal rearrangements involving the extracellular matrix^25^. Osteopontin, an extracellular matrix protein, is a prime example as it is up-regulated during the formation of glial scars after focal brain ischemia^45^. A receptor for osteopontin, the integrin αvβ3, is also up-regulated in reactive astrocytes that localize to the peri-infarct area 5 days after ischemia and to an osteopontin-rich, glial barrier 15 days post-ischemia, suggesting that αvβ3 and its extracellular ligands are involved in reactive astrogliosis^46^. In the study conducted by Lagos-Cabré and colleagues^47^, it was shown that αvβ3 integrin, beyond its migratory properties, regulates astrocyte reactivity. β3 integrin over-expression was sufficient to induce astrocyte reactivity and allow the glycoprotein Thy-1-induced migration, while ectopic control of β3 integrin levels modulated astrocyte responsiveness regardless of the state of inflammation. In regards to the present study, LPS-induced upregulation of αv, β3, β5, α5, and β1 integrin subunits was mitigated by LCT pre-treatment in astrocytes. In this study, we evidenced the boost in p-NfκB upon LPS stimulation alone in C8-D1A cells, which proves the activation of the NfκB pathway by LPS, resulting in over-production of inflammatory mediators. In addition, we observed phosphorylation from NfκB, with an attenuated signal intensity band regarding LCT itself and in conjunction with LPS Stimuli. These findings suggest that LCT may exert molecular mechanisms involving the modulation of these signaling pathways, particularly on NfκB protein activation.

Taken together, LCT decreased the levels of the pro-inflammatory cytokine IL-6, and chemokine CXCL10 in the LPS-induced reactive astrocytes. Additionally, this modulatory effect was associated with reduced activation of NfκB, along with a reduced expression of αvβ3 integrin especially .

Although the involvement of αvβ3 integrins in the inflammation process has been studied, alterations in integrin expression profiles remain strictly tied to developmental processes when studied in oligodendrocytes. Indeed, previous studies demonstrated that oligodendrocyte development is intricately linked to its extracellular environment, impacting all aspects of its progress, from migration and proliferation to growth, differentiation, and myelination^14^. Despite well-defined roles for integrin in proliferation and the differentiation of oligodendrocytes, its function in myelination remains largely unexplored. To this effect, the current study aims to demonstrate the direct involvement of integrins in the myelination process, by treating mature myelinating oligodendrocytes with a conditioned media from reactive astrocytes.

We were able to show that inflammatory conditions induced changes in integrin expression levels of mature oligodendrocytes. The expression levels of αv and β3 subunits were down-regulated, while β1 subunit expression was up-regulated. Our data indicate that levels of integrins were influenced by the inflamed medium, mirroring the pattern observed in astrocytes. This suggests that the dynamic expression and function of integrins in oligodendrocytes may be altered under conditions of neuroinflammation during demyelinating diseases.

Concerning β1 integrins, which are involved in oligodendrocyte differentiation and maturation, they have been shown to influence the myelination process^48^. Specifically, amyloid-β oligomers positively modulate MBP expression through their interaction with the integrin β1 receptor, Src-family kinase Fyn, and Ca^2+^/CaMKII effectors^48^. While β1 integrins are known for their role in oligodendrocyte differentiation and maturation, αvβ3 integrins are specifically involved in oligodendrocyte survival. The attachment of oligodendrocytes to fibronectin via αvβ3 integrin receptors was reported to render the cells ligation of αvβ3/fibronectin results in higher expression of activated Lyn kinase, leading to suppression of acid sphingomyelinase activity and preventing ceramide-mediated apoptosis^49^. Hence, it was put forth that increased interaction with αvβ3 receptors may prevent oligodendrocyte apoptosis.

We demonstrate that the expression levels of αv and β3 subunits were down-regulated, while β1 subunit expression was up-regulated in inflamed oligodendrocytes. Besides, results of MBP quantification, through western immunoblotting, showed a significant increase in expression level when oligodendrocytes were subjected to inflammatory conditions, whereas this overexpression was attenuated when cells were pre-treated with LCT. Notably, we observed that MBP levels returned to control values when integrin expression was modulated by the protein LCT; that specifically binds αv, β3 and β1 and blocks its activities. These results suggest that integrins participate in the regulation of MBP synthesis in inflammatory conditions. These results corroborate with those of Quintela-López and the team, which showed that integrin β1 (Itgb1) enhances MBP expression of oligodendrocytes treated with oligomers of beta-amyloid^48^. Similarly, they showed that treatment with antibody αCD29 (that specifically binds Itgb1 and blocks its activity) reduced Fyn phosphorylation^48^. Besides, the observed correlation suggests a potential explanation for the protective impact of LCT on oligodendrocytes exposed to an inflammatory milieu, as evidenced by the quantification of MBP through western immunoblotting.

The PI3K/Akt/mTor pathway plays an important role in promoting cell survival and inhibiting apoptosis^50,51^. The PI3K/AKT pathway is one of the protection mechanisms of cells against cell damage^52^. AKT is a serine/threonine kinase and the key mediated by the PI3K-initiated signaling, and it can promote cell survival^52,53^. PI3K activation results in the phosphorylation of Akt (p-Akt), and thus, p-Akt can be used as a marker of PI3K activation.

Besides its role in cell survival, the PI3K/Akt pathway is crucial for the myelination process in the central nervous system (CNS). Akt stimulates axonal wrapping and increases myelin thickness by activating the mTOR pathway. However, maintaining AKT activation can induce hypermyelination or demyelination^51,54^. Furthermore, studies have shown that the AKT signaling downstream effector, mTOR, is critically important for oligodendrocyte precursor cell (OPC) differentiation and myelination. Treatment with the mTOR inhibitor rapamycin in cell culture led to deficits in OPC differentiation, reduced expression of major myelin proteins and mRNAs, and CNS hypomyelination in developing mice, supporting the role of mTOR in myelination in vivo^55–57^.

In this study, we evaluated the phosphorylation levels of these effectors in stimulated oligodendrocytes treated with conditioned media (CM). Immunoblot analysis revealed that after 24 h, CM treatment led to decreased phosphorylation of mTOR, while increasing the phosphorylation of AKT. Conversely, when CM stimulated oligodendrocytes were exposed to LCT at 100 nM, there was a reduction in phosphorylated AKT and an increase in the phosphorylation of mTOR, without altering their basal expression levels. Our findings indicate that slight downregulation of αvβ3 integrin results in Pi3K and mTor inhibition and AKT activation in response to cellular damage induced by CM. Interestingly, LCT modulates this signaling pathway, indicating the possibility that LCT protects oligodendrocytes from CM-induced injury by the activation of the PI3K/Akt pathway associated to the demyelination.

The findings from in vitro experiments were subsequently validated in an animal model, employing cuprizone as a demyelinating agent. The toxic demyelination model induced by cuprizone is commonly used to study the process of de- and remyelination in vivo. The cuprizone mechanism of action is not fully understood, but it is known to combine energy failure and increased production of reactive oxygen species as a consequence of mitochondrial damage^58^, leading to oligodendrocyte cell death. This disturbance in the CNS microenvironment leads to the recruitment of microglial cells. It was shown that accumulated microglial cells in the lesion areas derive from activated and proliferating resident cells, as well as cells attracted from adjacent, non-affected brain regions^59^. In our study, the histopathological analysis showed that cuprizone induced the activation and recruitment of immune cells to the inflammation site in the mice’s brain as shown by hematoxylin-eosin staining. We also used CD3 antibody immunostaining on mouse brain sections to identify T cells. In mice that developed demyelination, we observed a clear presence of infiltrating T cells in the brain (Fig. 4). The presence of CD3-positive T cells suggests that these cells are proliferating locally within the brain, or migrating from peripheral lymphoid organs because cuprizone does affect the blood-brain barrier integrity^60^. Histological analysis revealed that LCT reduced neuroinflammation, as evidenced by decreased inflammatory cell infiltration in the brains of CPZ mice. This effect may be linked to increased remyelination, as indicated by the presence of more myelinated areas. Additionally, LCT decreased the expression of LTCD3 + cells in CPZ mice and promoted their re-localization by inhibiting their migration across the blood-brain barrier (BBB).

In the cuprizone model, it was shown that demyelination was induced in the *corpus callosum*, cerebral cortex, striatum, and cerebellum^61^. Demyelination persists during continuous cuprizone feeding but stops when cuprizone treatment is halted, allowing for spontaneous remyelination^62^. However, spontaneous remyelination fails to compensate for demyelination, resulting in chronic axonal injury and permanent neurological disability. Failure of myelin repair may be due to inadequate or insufficient recruitment of OPCs or to a failure of recruited OPCs to differentiate into remyelinating oligodendrocytes^63^. Demyelinated axons may be unresponsive to remyelination because of intrinsic axolemmal alterations such as the expression of inhibitory cell surface molecules (like polysialylated neural cell adhesion molecule PSA-NCAM), neurofilament fragmentation, or energy failure^64^.

The significant level of remyelination detected through LFB staining in LCT-treated mice, compared with the ones undergoing SR, proves the ability of integrin-modulator LCT to promote remyelination. This level of remyelination positively impacted behavioral studies performed on the mice. The cuprizone model generally translates into motor and behavioral deficiencies in mice, which was first reported by Morell and his colleagues^65^. Cuprizone-treated animals displayed a lower activity level compared to control animals. Furthermore, C57BL/6 mice treated with cuprizone developed motor dysfunction after 5 weeks, this dysfunction generally persists for a prolonged period of time after withdrawal of the toxic agent^66^. In this context, mice treated with LCT after the cuprizone administration period showcased a decrease in immobility and improved limb coordination.

Overall, these results strongly suggest a potential role of integrin-modulator LCT in promoting axonal remyelination. LCT may have affected the oligodendrocytic population by (i) inducing the heightened survival of mature oligodendrocytes through increased interaction with αvβ3 integrins, (ii) or the differentiation of OPCs into mature myelinating oligodendrocytes via interacting with the β1 integrins expressed by OPCs^67^, as these integrins were shown to be implicated through in vitro experiments; (iii) or indirectly by influencing astrocyte production of differentiation factors for OPCs^68^. The latter requires a more in-depth study.

## Conclusion

**Fig. 5:**
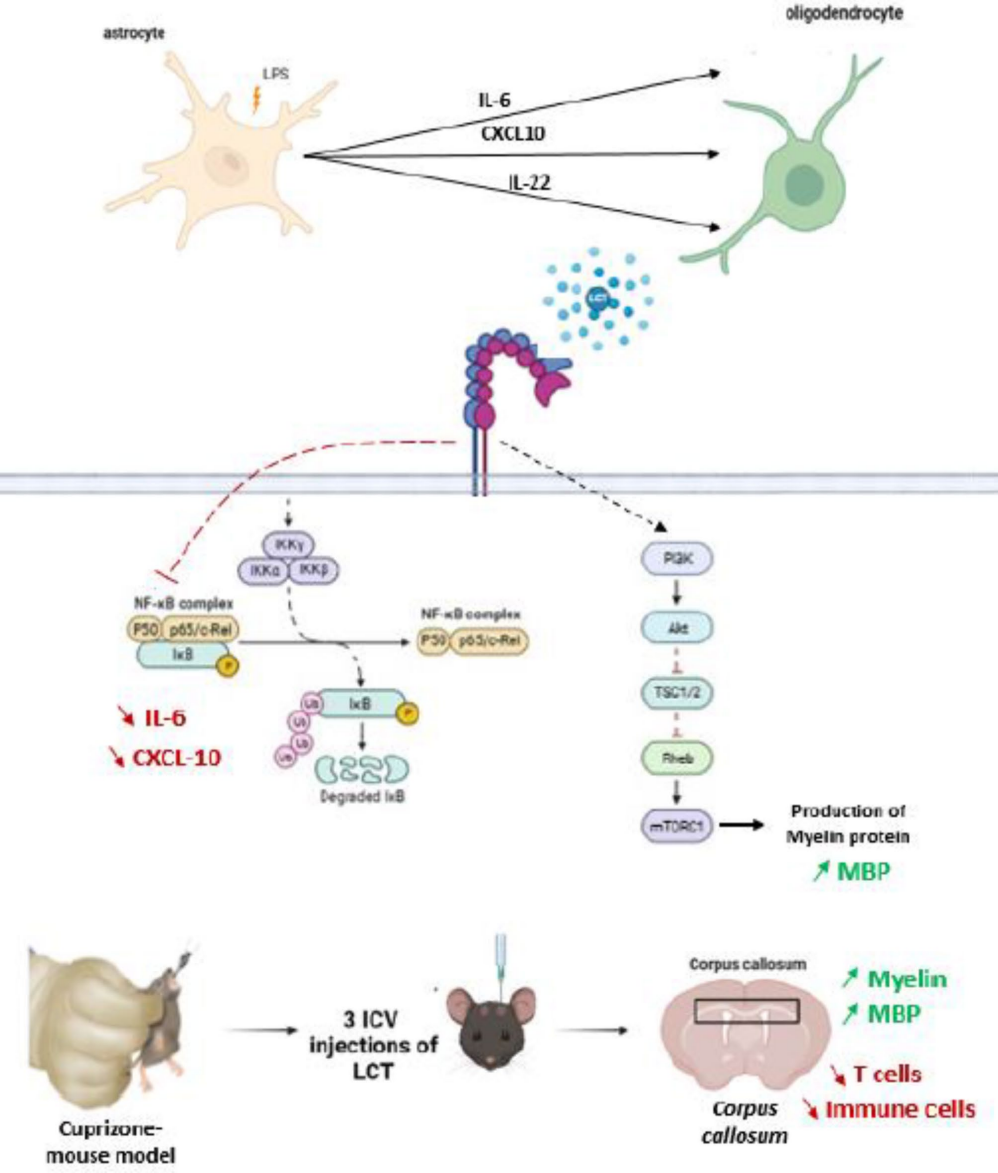
Graphical summary from cellular and animal model studies to demonstrate the role of integrin modulator to regulate effectors involved in neuroinflammation and demyelination.

In conclusion, the present study highlighted the significance of the involvement of αvβ3 and β1 integrins in not only the inflammatory processes associated with the NfκB pathway modulation but also the myelination associated to PI3K/mTor/AKT pathway modulation (Fig. 5). Specifically, the modulation of αvβ3 integrins via LCT, as an integrin antagonist, correlated with reduced pro-inflammatory cytokines and enhanced remyelination. These findings suggest a promising avenue for future research, exploring the intricate interplay of integrins in neuroinflammation and myelin repair. In keeping with this, integrin modulators, whether of natural or synthetic origins, offer a therapeutic avenue worth exploring. Further investigations into the underlying molecular mechanisms and clinical applications of integrin modulation may contribute to the development of novel therapeutic strategies for conditions involving demyelination, such as Parkinson disease, and Multiple Sclerosis.

## Methods

### Biological material

Lebecetin (LCT) from the tunisian viper *Macrovipera lebetina transmediterranea* (*M. lebetina*) was purified as previously described by Sarray et al.,^69^. Purified LCT was analysed via total protein staining, UPLC and Mass Spectrometry to ascertain the purity of the molecule, LCT was resuspended in PBS and quantified then stored as single use aliquots at – 20 °C until needed.

### Study of in vitro models

#### Cell lines and cell culture

Rodent astroglial (C8-D1A, Ref: CRL-2541, American Type Culture Collection (ATCC), Manassas, VA, USA) and oligodendroglial (158 N)^70^ cell lines were graciously provided by Dr. José Luis from the Institute of Neurophysiopathology (Aix-Marseille University, Faculty of Pharmacy, Marseille, France) and Pr. Gérard Lizard from the Bio-peroxIL laboratory (Dijon, France), respectively. Cell lines were grown in DMEM culture media, with units/ml of Penicillin and Streptomycin, and 10% (v/v) heat-inactivated Fetal Bovine Serum (FBS). Cells were maintained at 37 °C in a humidified atmosphere of 5% CO_2_ and passaged biweekly.

#### Reagent preparation

LPS solution 500× (#00-4976-93, eBioscience™) was diluted to 1 mg/ml in PBS, aliquoted and stored at – 20 °C. The solution was then brought to working concentrations with the adequate culture medium.

#### Cell viability assessment

Cell viability was evaluated by 3-(4,5-dimethylthiazol-2-yl)-2,5-diphenyltetrazolium bromide (MTT) reduction assay. C8-D1A and 158 N cells were respectively seeded at a density of 5 × 10^3^ cells per well in 96-well tissue culture plates and allowed to grow overnight at 37 °C. Cells were cultured for 24 h in the absence or presence of different concentrations of lebecetin. Cell viability was assessed by MTT (3-(4, 5-dimethylthiazol-2-yl)-2, 5-diphenyltetrazolium bromide) assay as previously described (21). Absorbance at 560 nm was measured using a Multiskan SkyHigh Microplate reader (ThermoScientific). The cell viability was expressed as percentage of the viable cells relative to control.

#### Astrocyte treatment and LPSinduced inflammation assay

C8-D1A cells were seeded in 6-well plates (6 × 10^5^ cells/well), then treated with LPS (500 ng/ml) and LCT (100 nM), both individually and in combination, in which case LCT was introduced 3 h prior to the addition of LPS. Control group with no treatment was considered. Cell supernatants were collected by centrifugation at 1500 rpm for 5 min for quantitative analysis of cytokines. The cells kept on the bottom of the wells were processed for whole-cell lysate collection for subsequent western immunoblotting assays.

#### ELISA quantification of cytokines in reactive astrocytes

24 h later, the culture media was collected for ELISA quantification of released cytokines and chemokines. Classic ELISA was performed for the quantification of cytokines and chemokine (IL-6 IL-22, and CXCL10) using the Mouse IL-6 Duoset ELISA (#DY406, RandD systems, Minnesota, USA), the Mouse IL-22 Duoset ELISA (#DY582, RandD systems, Minnesota, USA) and the Mouse CXCL10/IP-10/CRG-2 DuoSet ELISA (#DY466, RandD systems, Minnesota, USA), based on the manufacturer’s protocol. Optical Density was determined using a microplate reader set to 450 nm (MultiskanSkyHigh, Thermo Scientific). Each sample was analyzed in triplicate.

#### Integrin expression and signaling pathways regulation in reactive astrocytes

Cell lysate collected from astrocytes previously treated with LPS and/or LCT was used to quantify the expression of integrins, as well the expression of total and phosphorlated NfκB through Western Immunoblotting.

Treated cells were lysed and aliquoted, protein quantification was performed with a BCA assay (#QPBCA, QuantiPro™ BCA Assay Kit, Sigma-Aldrich). Protein samples were then resolved onto an SDS-PAGE 4–12% (Mini-PROTEAN Tetra Vertical Electrophoresis Cell, Bio-Rad, USA), then transferred to a PVDF membrane (Western blot blotting tank omniBLOT, Cleaver Scientific, UK). Membranes were blocked with 5% BSA solution then incubated overnight at 4 °C with primary antibodies anti-αv (1:1000, Abcam, #ab179475), α5 (1:1000, Abcam, #ab150361), β1 (1:1000, Abcam, #ab179471), β3 (1:1000, Abcam, #ab119992), and β5 (1:1000, Abcam, #ab309092) integrin subunits, anti-NfκB p65 (1:500, Abcam, #ab32536) and anti-NfkB p65 (phospho-S468) (1:500, abcam, #ab32536), anti-β-actin (1:1000, Abcam, #ab8226), or anti-vinculin (1:1000, Cell Signaling, #4650). Membranes were then washed with PBST (0.05% Tween 20) before incubation with secondary HRP-conjugated secondary anti-mouse (1:10,000, Promega, #W4021) or anti-rabbit (1:1000, Promega, #W4011) antibodies. Bands were revealed with the use of Luminol-based ECL substrates (ECL western blotting substrate kit (#ab65623, abcam) or High Sensitivity ECL substrate kit (#ab133406, abcam), Western blots of integrin subunits were revealed using a classic developer and dark room technique, films were dried and scanned at 300 dpi resolution, while western blots of transcription factors NfkB and phosphorylated NfkB were imaged using the ChemiDocTM Imaging System (#12003153, Bio-Rad, USA) quantitative analysis of all western blots was performed using the ImageJ software.

#### Treatment of mature oligodendrocytes with astrocyte’ conditioned media (CM)

**Fig. 6:**
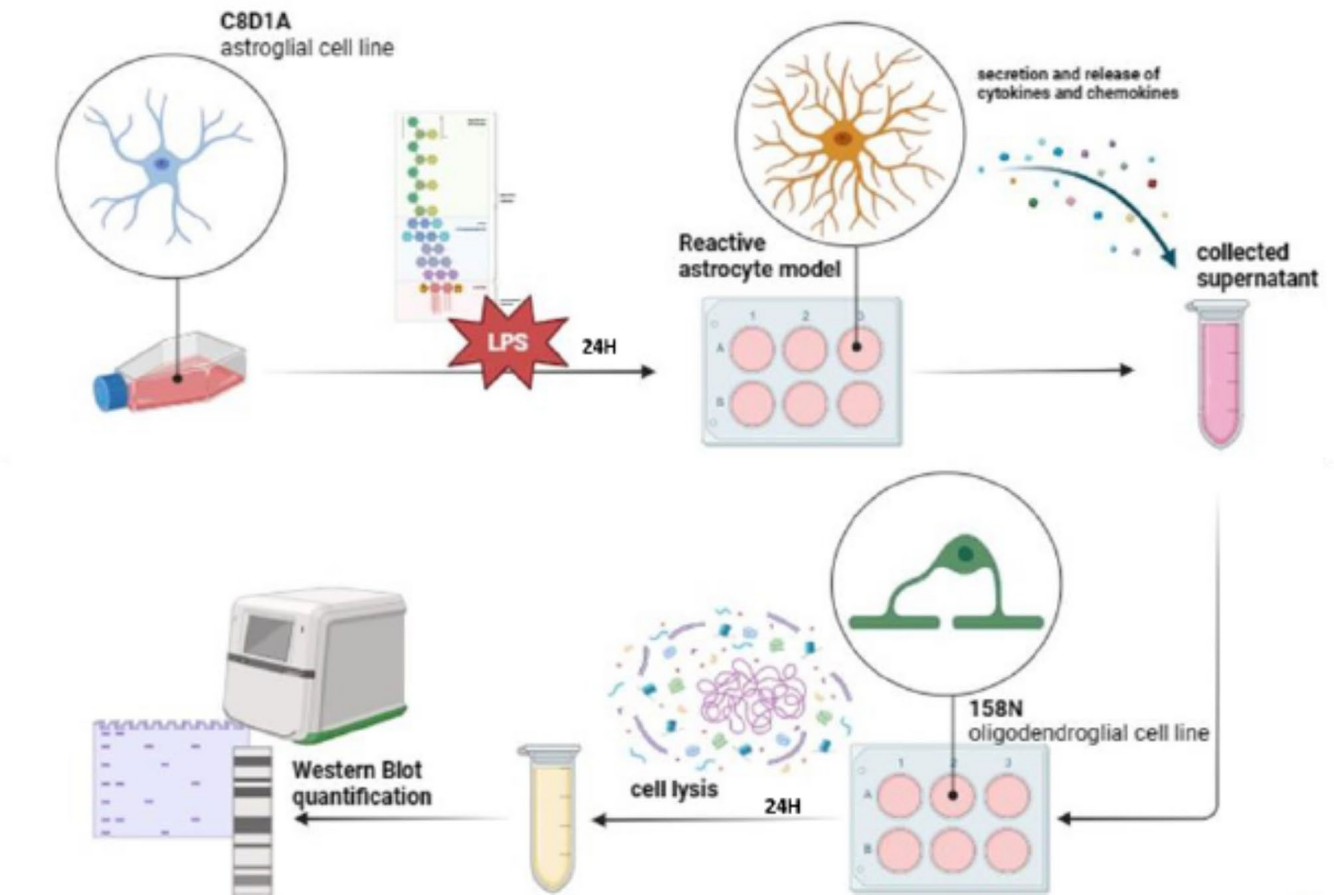
Graphical timeline of the preparation of conditioned media from reactive astrocytes, and its use in the treatment of mature oligodendrocytes.

To further understand the impact of effectors released by reactive astrocytes on the myelination process, an astro-oligodendroglial indirect and unilateral “co-culture” system was established. Astrocytes were plated on a 6-well plate (6 × 10^6^ cells/well), then treated with LPS (500 ng/ml), 24 h later, CM was transferred to oligodendrocytes similarly plated on a 6-well plate (6 × 10^6^ cells/well), and pretreated with LCT (100 nM). Both cell types were lysed with Laemmli buffer 24 h following their respective treatments, and stored at – 20 °C for Western Immunoblotting assays (Fig. 6).

#### Determination of protein expression and signaling pathways regulation in stimulated oligodendrocytes

Cell lysate from treated oligodendrocytes was used to quantify the expression of Myelin Basic Protein (MBP), integrin subunits, as well as transcription factors PI3K, AKT and mTOR via Western Blot.

Protein quantification was performed with a BCA assay (#QPBCA, QuantiPro™ BCA Assay Kit, Sigma-Aldrich). Protein samples were then resolved onto an SDS-PAGE 4–12% (Mini-PROTEAN Tetra Vertical Electrophoresis Cell, Bio-Rad, USA), then transferred to a PVDF membrane (Western blot blotting tank omniBLOT, Cleaver Scientific, UK). Membranes were blocked with 5% BSA solution then incubated overnight at 4 °C with primary antibodies anti-Myelin Basic Protein (MBP, 1:2000, RandD systems, #MAB42282), anti-αv (1:1000, Abcam, #ab179475), α5 (1:1000, Abcam, #ab150361), β1 (1:1000, Abcam, #ab179471), and β3 (1:1000, Abcam, #ab119992), and β5 (1:1000, Abcam, #ab309092) integrin subunits, anti-PI3K p85 (1:500, Abcam, #ab191606), anti-pan-AKT (1:1000, Cell Signaling, #4691P), anti-AKT (phospho S473) (1:1000, Cell Signaling, #4060T), and anti-mTOR (phospho S2448) (1:500, Abcam, #ab109268), anti-β-actin (1:1000, Abcam, #ab8226), or anti-vinculin (1:1000, Cell Signaling, #4650). Membranes were then washed with PBST (0.05% Tween 20) before incubation with secondary HRP-conjugated secondary anti-mouse (1:10,000, Promega, #W4021) or anti-rabbit (1:10,000, Promega, #W4011) antibodies. Bands were revealed with the use of Luminol-based ECL substrates (ECL western blotting substrate kit (#ab65623, abcam) or High Sensitivity ECL substrate kit (#ab133406, abcam), Western blots of integrin subunits were revealed using a classic developer and dark room technique, films were dried and scanned at 300 dpi resolution, while western blots of transcription factors PI3K, pan-AKT and phosphorylated AKT, and mTOR were imaged using the ChemiDocTM Imaging System (#12003153, Bio-Rad, USA) quantitative analysis of all western blots was performed using the ImageJ software.

### Study of in vivo model

#### Animal care

Female C57BL/6 mice were used for the induction of an animal model of demyelination. Mice were bred and provided by the Pasteur institute’s animal unit with a requested age of 6–8 weeks, and weight averaging between 20 g and 25 g. The animals were randomly split into 2 groups and housed in polycarbonate cages with sawdust bedding, situated in environmentally controlled rooms (22 ± 2 °C and 50 ± 10% humidity) alternating between 12 h periods of light and darkness, starting at 7 a.m.

In addition, animals underwent routine cage maintenance twice a week (every Monday and Thursday at 9 a.m.), and provided food (standard pellet diet) and clean drinking water ad libitum.

The animal management and experimental procedures of this study were conducted in accordance with the ARRIVE guidelines (https://arriveguidelines.org*)* and approved by biomedical ethics committee of the Pasteur Institute of Tunis, Tunisia (Number 2022/3/I) prior to the experimentation. All experimental methods were carried out in accordance with relevant guideline and regulations.

#### Development of the cuprizone mouse model of demyelination

**Fig. 7:**
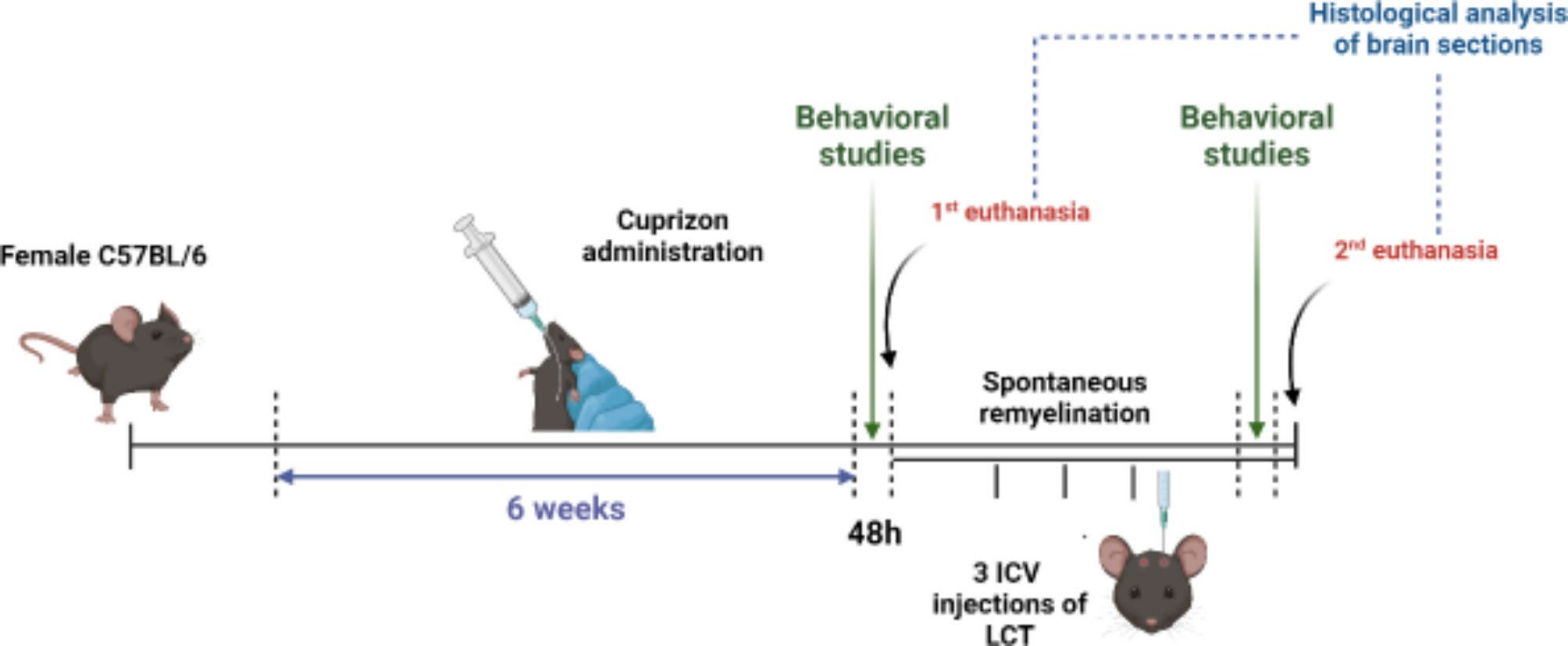
Graphical timeline of the development of cuprizone model of demyelination followed by LCT treatment or spontaneous remyelination.

Cuprizone solution for in vivo purposes was freshly prepared each morning, by adding cuprizone powder to corn oil for a final concentration of 300 mg/kg of body weight. After a week of acclimation, the cuprizone/corn oil mixture was fed to the mice via oral gavage (Fig. 7). This process took place on a daily basis over a period of 6 weeks. Similarly, the vehicle group was administered a daily regimen of corn oil through oral gavage for the duration. Following the 6-week period of cuprizone administration, behavioral studies were carried out, in conjunction with daily observation over the last 6 weeks, in order to validate the successful onset of the experimental model (Fig. 7). 48 h later, the vehicle group labeled CTR (−) (*n* = 6) as well as the positive control group labeled CPZ (*n* = 6) were euthanized and their brains were collected for further testing (Fig. 7).

The remaining mice were divided into two groups. The first group, labeled SR (*n* = 6), experienced spontaneous remyelination, while the second group, labeled LCT (*n* = 6), underwent a treatment phase lasting two weeks. In this treatment phase, the mice received a total of three intracerebroventricular injections, each delivering 5 µg (1 mg/ml) of LCT directly into the brain (Fig. 7). These injections were spaced apart with intervals of 5 days to allow for recovery, and all efforts were made to minimize animal suffering. Following the treatment phase, behavioral studies were conducted again for comparison. Subsequently, after 48 h, the mice were euthanized, and their brain tissue was collected for further testing (Fig. 7).

#### Behavioral studies

The inverted screen test was used to measure motor strength and coordination of the limbs. The mice were individually placed in the center of the screen; the latter was then rotated to an inverted position with the mouse’s head declining first. The test was recorded until the mouse fell off or until the criterion time of 60 s was reached, in which case the mouse was manually removed from the screen. The results were scored as follows: Falling between 1 and 10 s = 1 ; Falling between 11 and 25 s = 2 ; Falling between 26 and 60 s = 3; Persisting after the 1060 s mark = 4^71^.

For the tail suspension test, mice were carefully suspended by their tails above a padded surface in a position where they could not escape nor hold on to nearby surfaces, a measured length of tape was wrapped around the tail of the mouse on one end, approximately 5 mm away from the tip, and the other end was attached to an elevated bar. The resulting escape-oriented behaviors were quantified for a period of 6 min, these behaviors include attempting to reach the surrounding apparatus walls or suspension bars, strong shaking of the body, or movement of the 4 limbs which is similar to running^72^.

#### Tissue staining

Mice were euthanized through rapid decapitation. Brains were removed from all groups, post-fixed in 10% paraformaldehyde, and embedded in paraffin. For light microscopy, 7 μm serial coronal paraffin sections between bregma − 0.82 mm and bregma − 1.94 mm were cut according to the mouse atlas by Paxinos and Franklin^73^. The cuts were made with a rotary microtome (RM2245, Leica, Wetzlar, Germany) and evaluated microscopically. Brain sections of mice from different treatment groups were stained using Luxol Fast Blue (LFB) in order to determine the level of demyelination. LFB stains myelin and myelinated axons blue whereas the counterstaining with CresylEcht Violet al.lows for Nissl bodies to be colored violet on formalin-fixed tissue. A number of brain sections from each group were dedicated to staining with Hematoxylin and Eosin (HE) (Sigma, St. Louis, MO, USA) in order to assess the degree of neuroinflammation. The remaining sections were used to study the expression of the Myelin Basic Protein (MBP) using mouse anti-MBP antibody (Abcam, #ab209328) and inflammation using anti-CD3 antibody (Abcam, #ab16669) by immunohistochemistry. The determination of peroxidase activity was realized using 3,3′-Diaminobenzidine (DAB) chromogen. Stained brain sections were all observed under an optical microscope and pictures were snapped at random intervals (Olympus BX51 microscope mounted with an Olympus DP12 digital camera).

### Statistical analysis

All data were normally distributed and presented as the mean ± standard deviation (mean ± SD). In cases of multiple comparisons, data were analyzed using a one-way analysis of variance (one-way ANOVA) followed by Tukey’s test for multiple comparisons by GraphPad Prism 8.0 software. A *p* value of less than 0.05 was considered statistically significant.

## Electronic supplementary material

Below is the link to the electronic supplementary material.

Supplementary Material 1

## Data Availability

The datasets used and/or analyzed during the current study are available from the corresponding author on reasonable request.
