# Supplementary material for: Modulation of αv integrins by lebecetin, a viper venom-derived molecule, in experimental neuroinflammation and demyelination models
Source: Sci Rep. 2024 Sep 27;14:22398. doi: 10.1038/s41598-024-73259-1 (PMC11436777; doi:10.1038/s41598-024-73259-1)

**Supplementary material**

**Figure 1 :** cell viability **in (A)(B)** astrocytes (C8-D1A) and **(C)** oligodendrocytes.

**A**

**C**

**B**

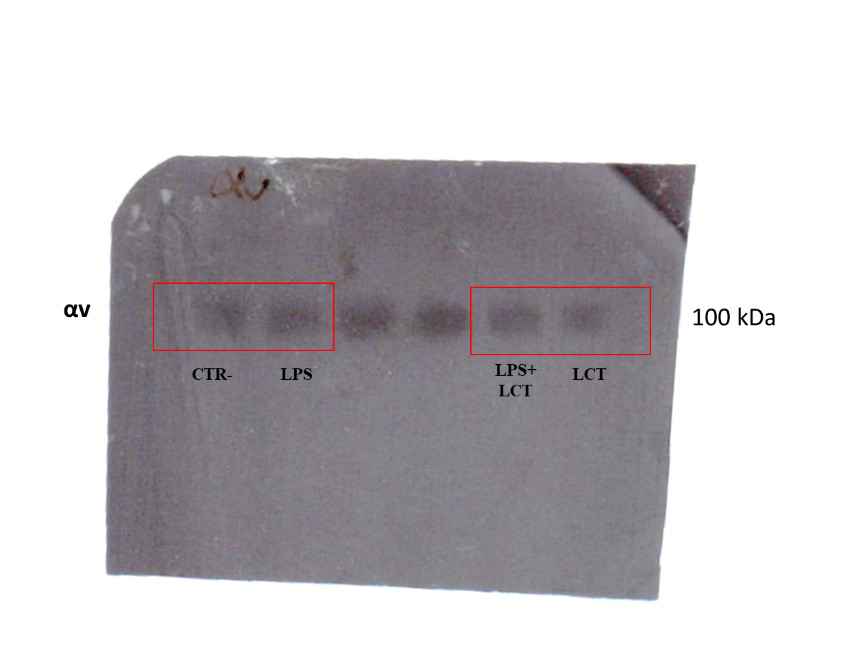
**Figure 2 :** Original western blots for quantification of integrin subunits at loading control in C8-D1A (fig.1D):


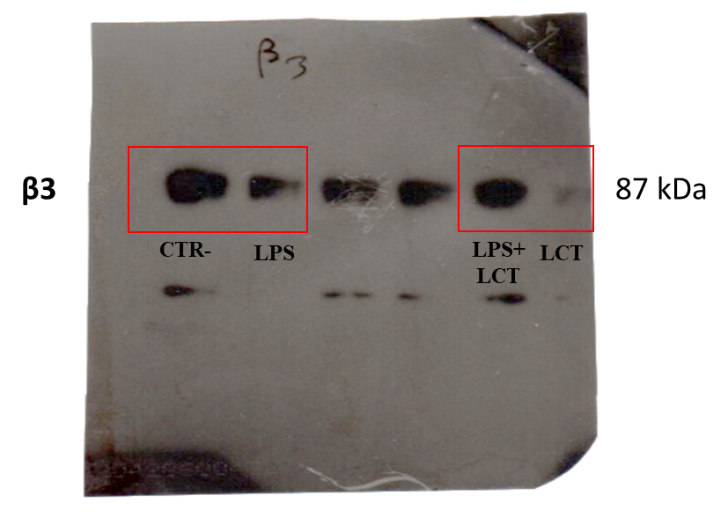


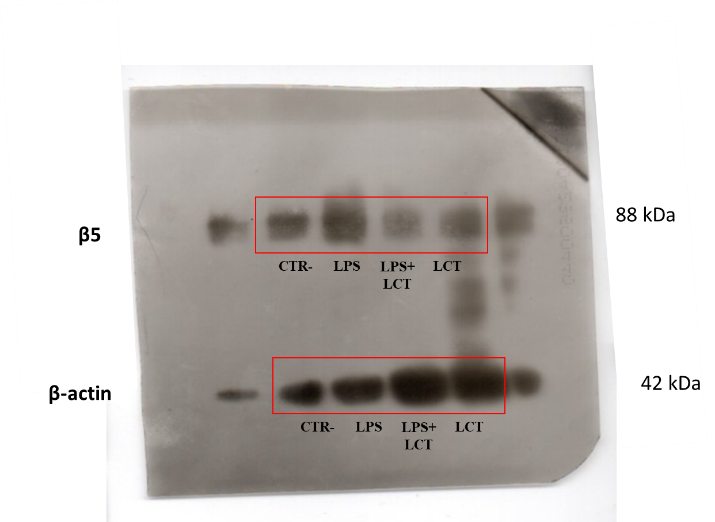

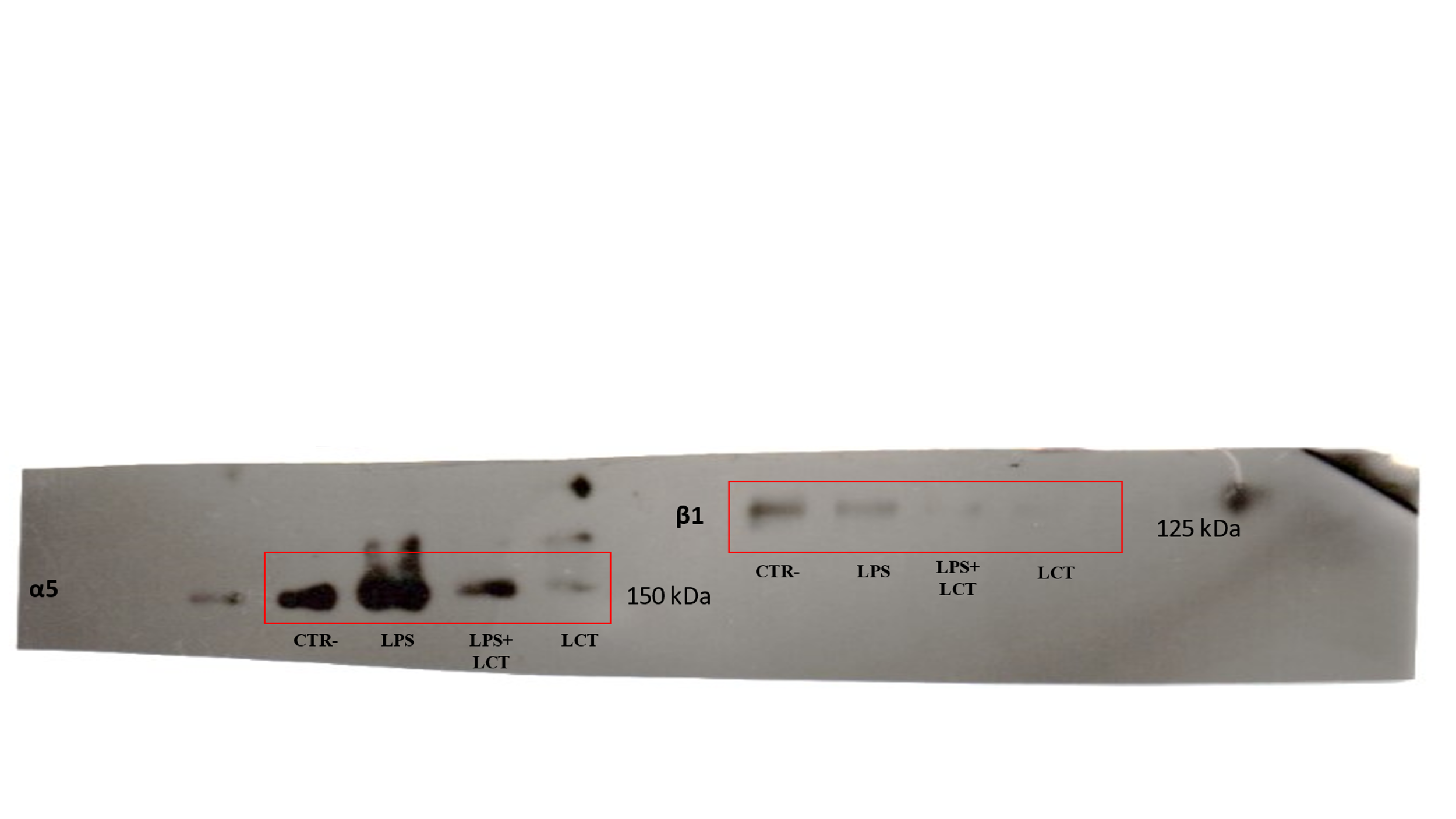


**Figure 3 :** Original western blots for quantification of NfkB p65 and p-NfkB p65 with loading control in C8-D1A (fig.1J)


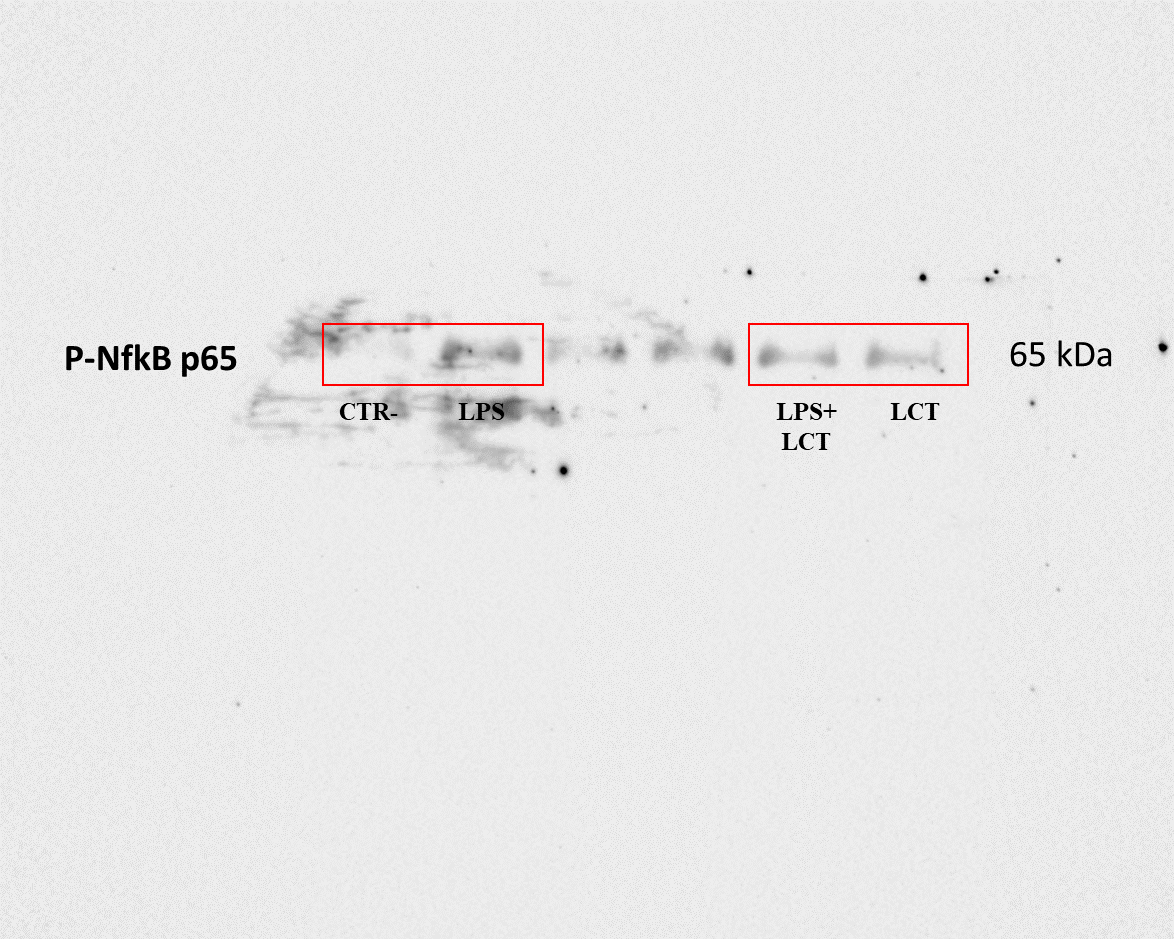

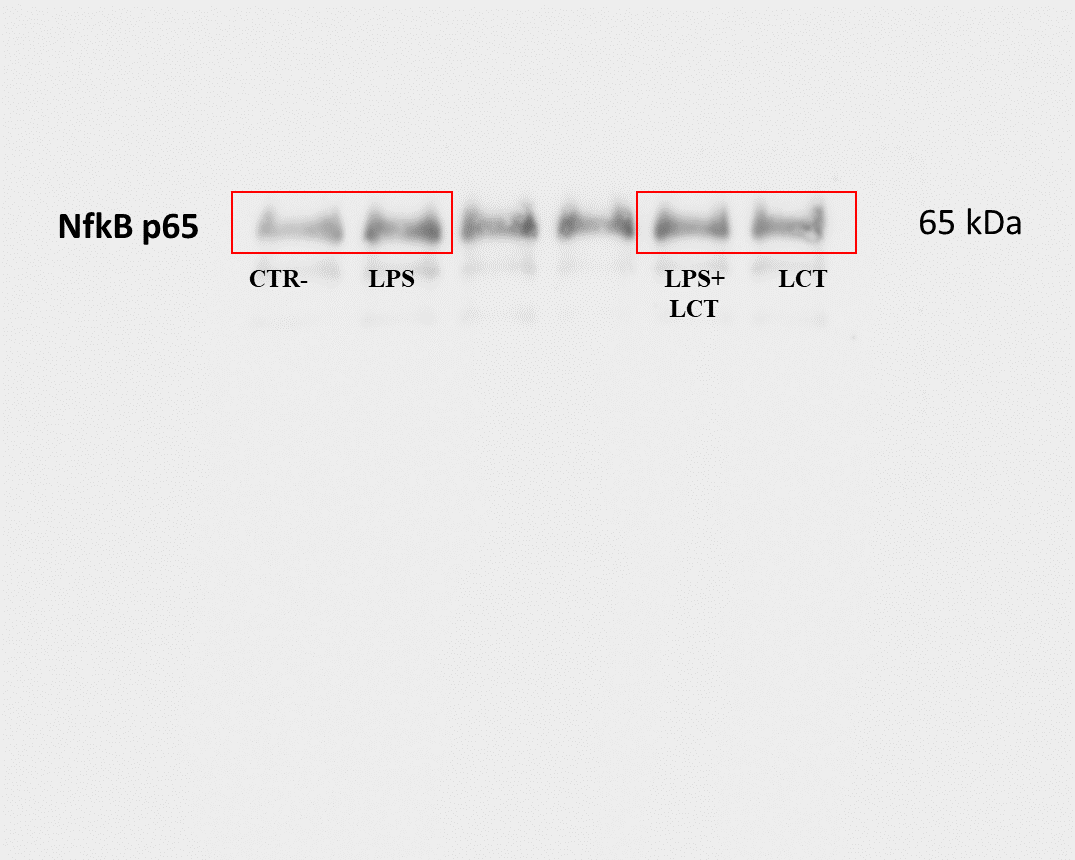


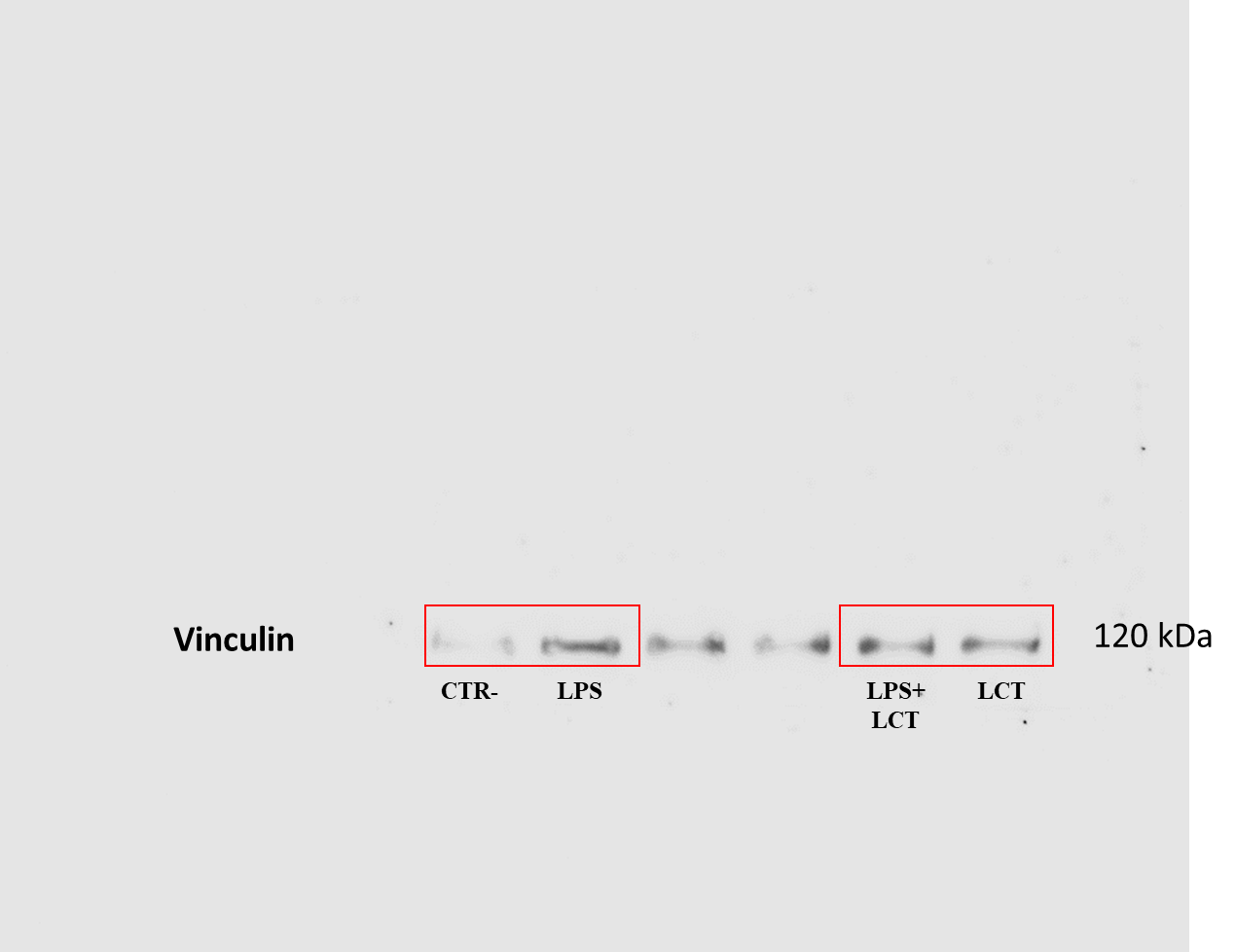


**Figure 4 :** Original western blots for quantification of MBP with loading control in 158N (fig.2A)


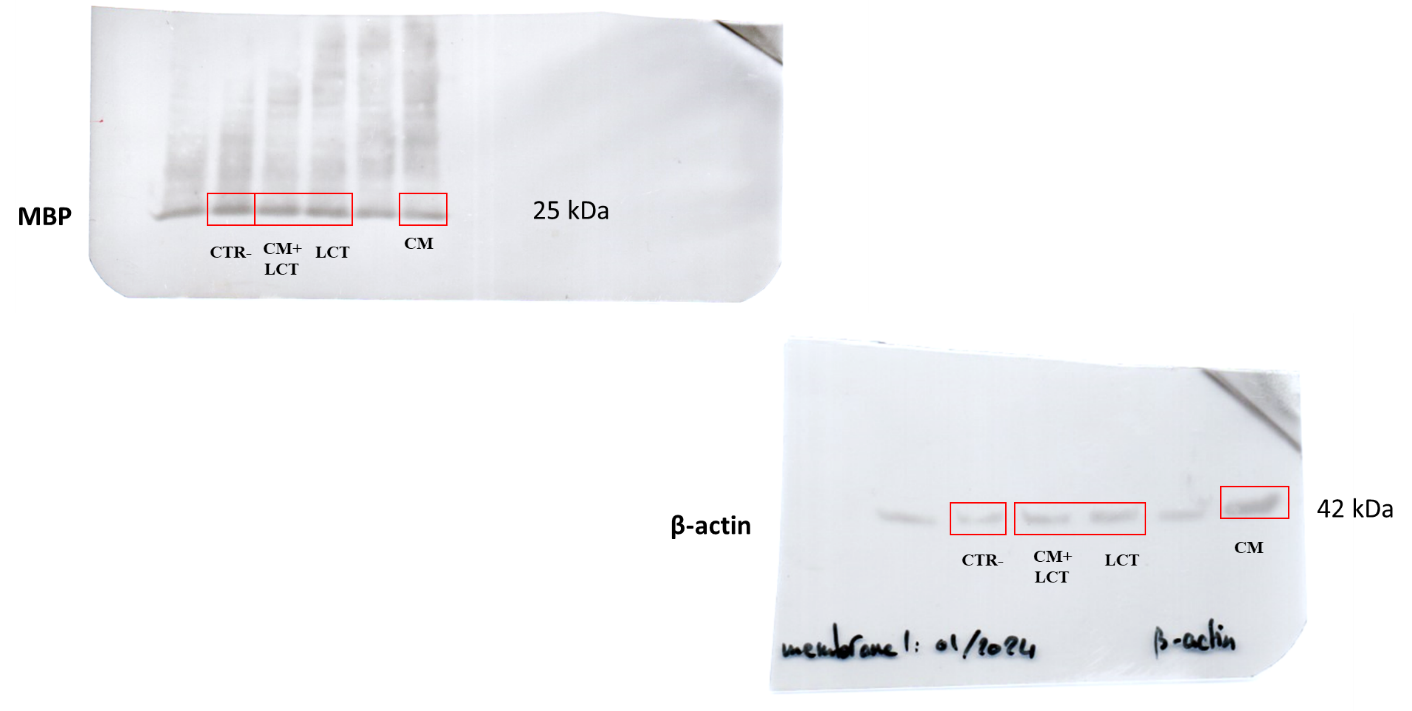


**Figure 5 :** Original western blots for quantification of integrin subunits and loading control in 158N (fig.2C)


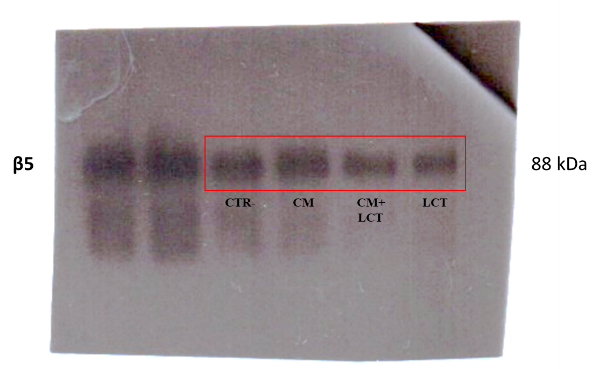

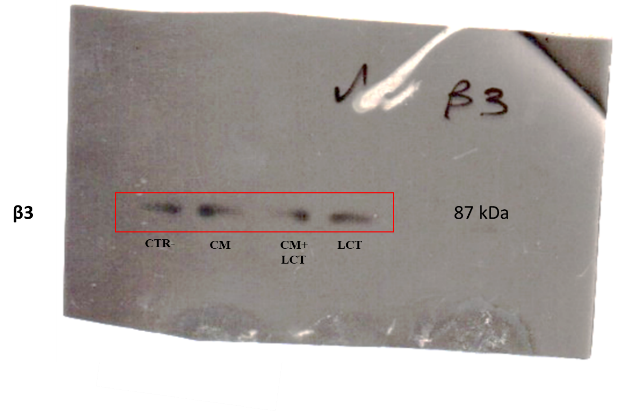

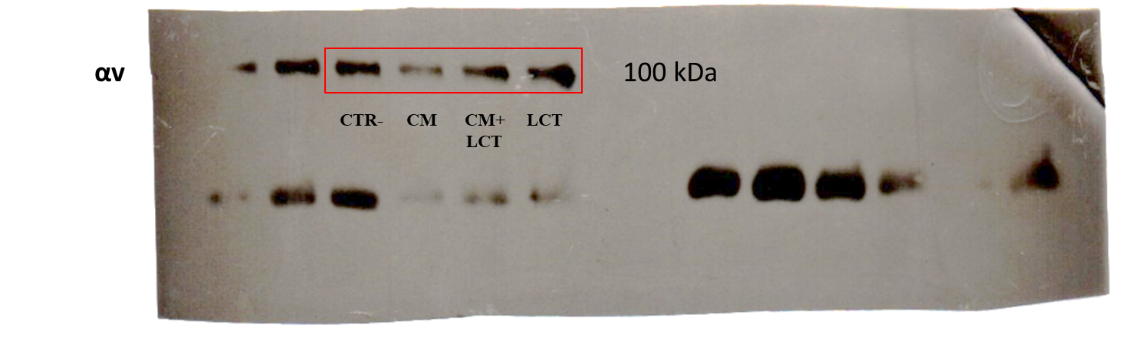


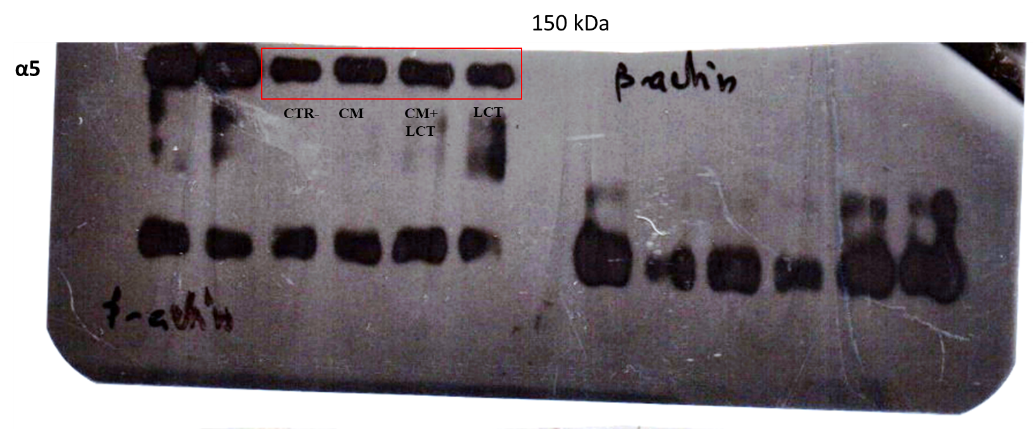

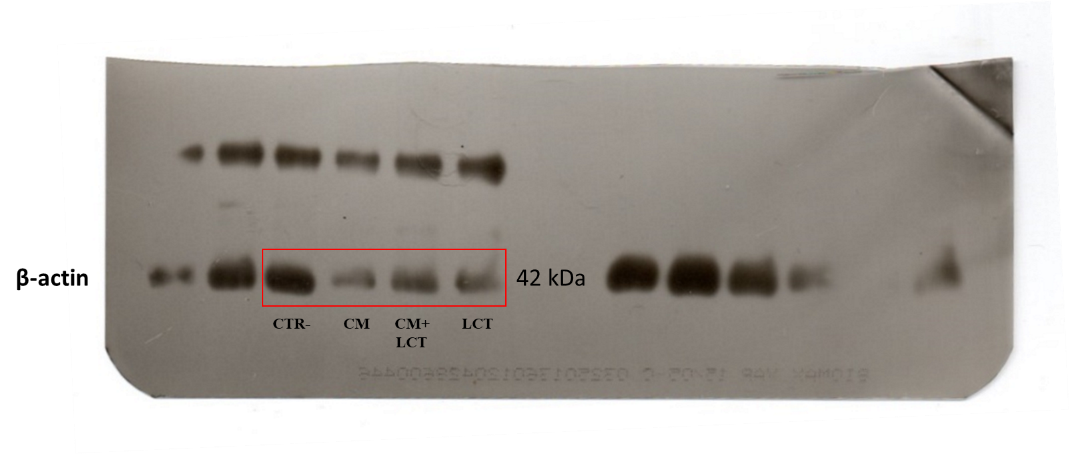

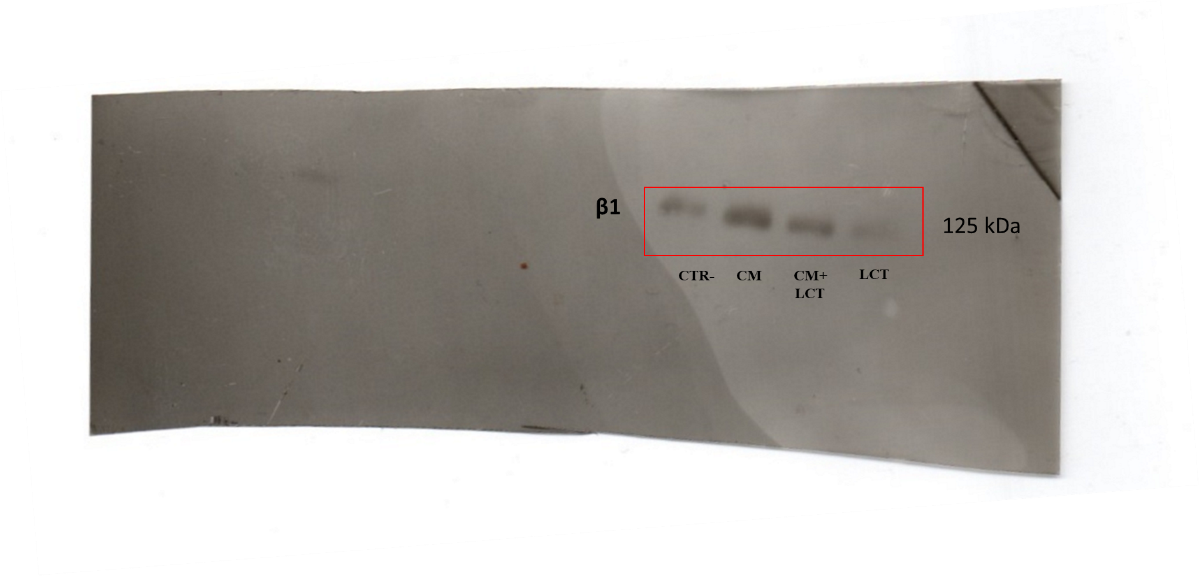


**Figure 6 :** Original western blots for quantification of PI3K, pan AKT and p-AKT, and p-mTOR with loading control in 158N (fig.3A)


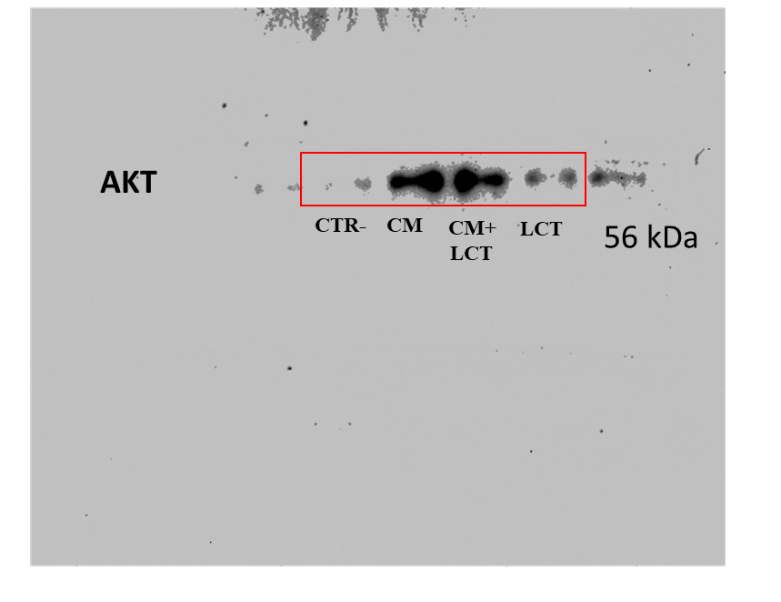

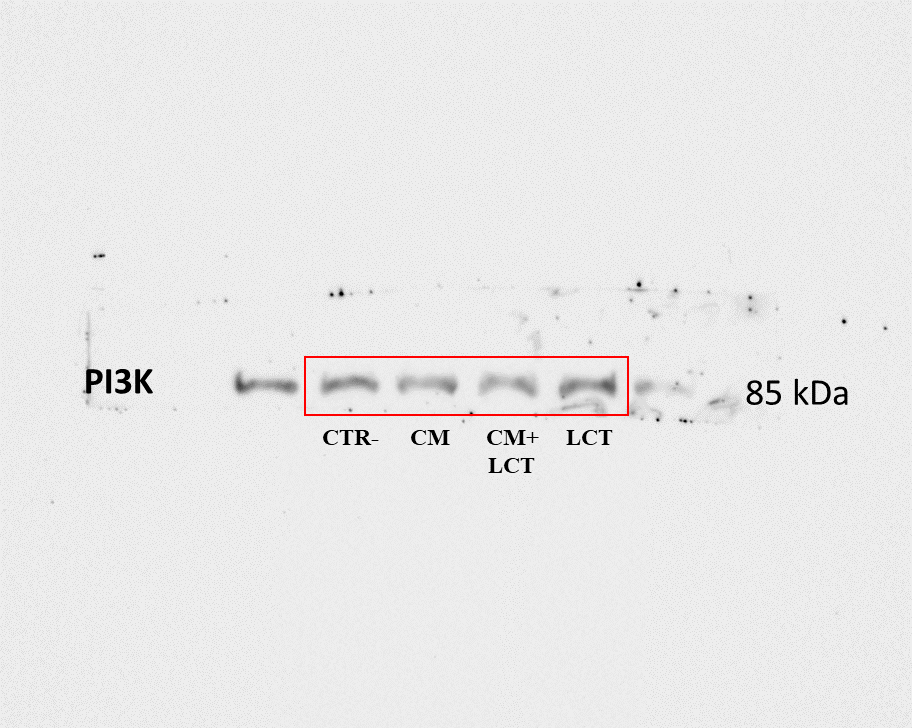


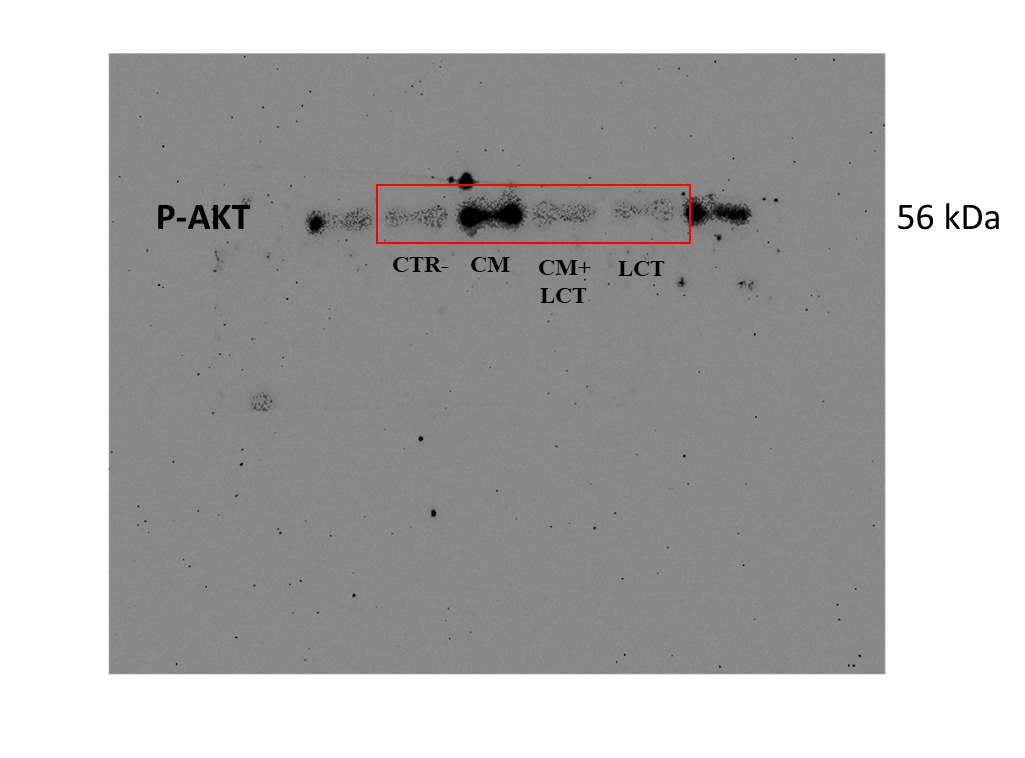


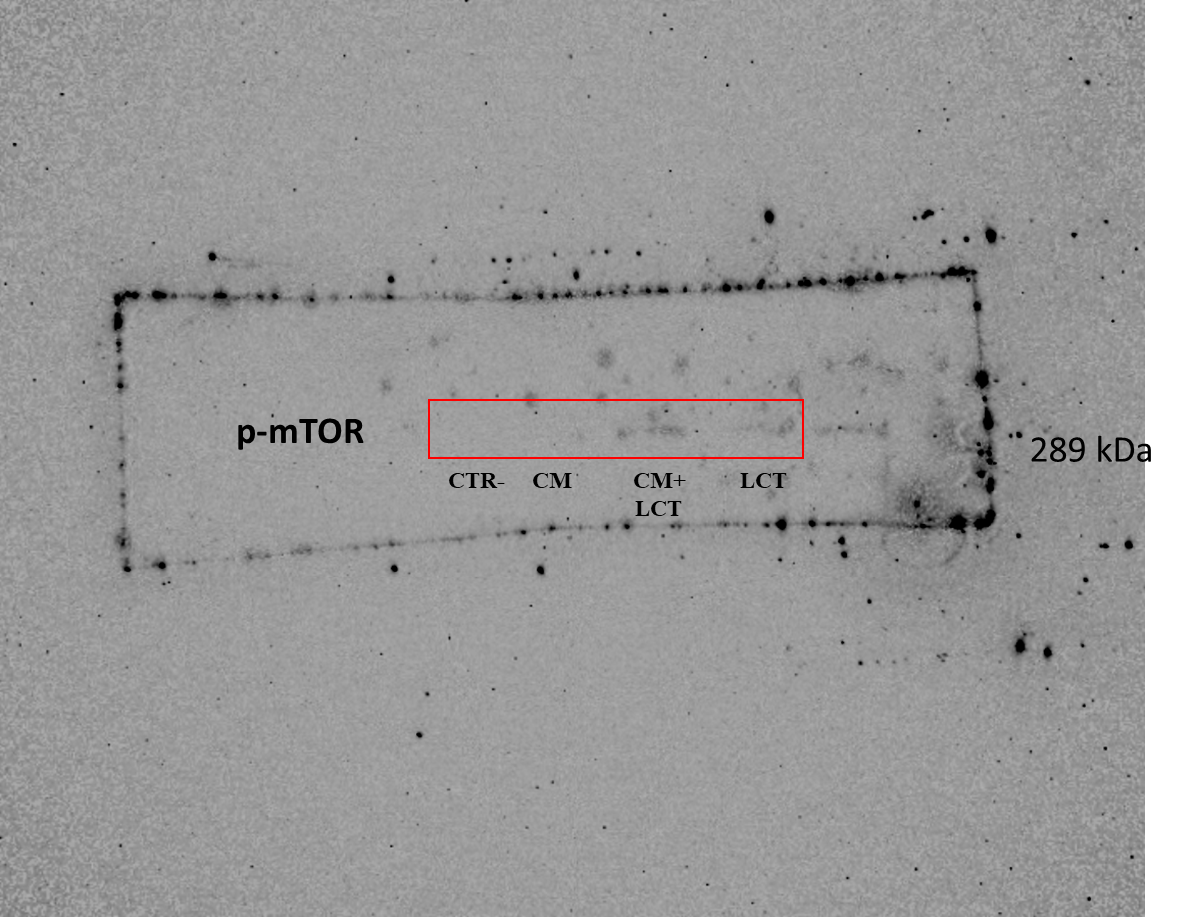


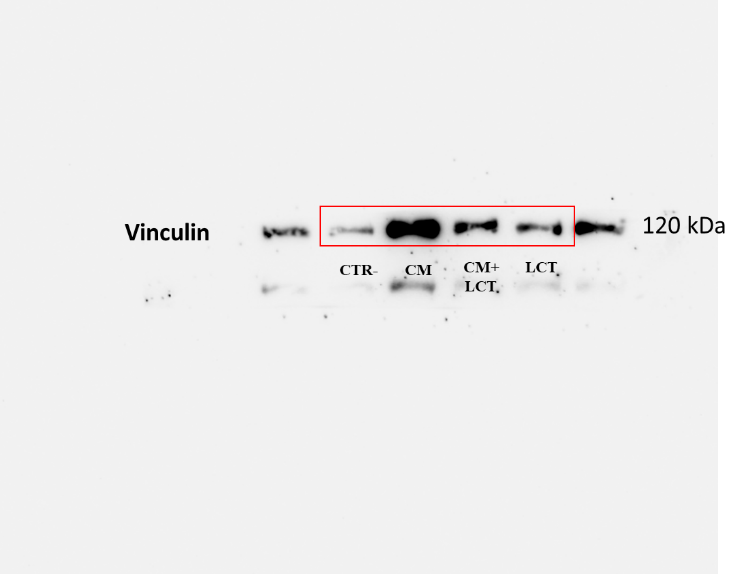

Supplement: Supplementary file 1 — Supplementary Material 1 [file 41598_2024_73259_MOESM1_ESM.docx]
